# Supplementary material for: Association of hyperuricemia with coronary heart disease and other cardiovascular outcomes: A systematic review and dose-response meta-analysis
Source: PLoS One. 2025 Nov 18;20(11):e0337091. doi: 10.1371/journal.pone.0337091 (PMC12626327; doi:10.1371/journal.pone.0337091)

# Association of hyperuricemia with coronary heart disease and other cardiovascular outcomes: A systematic review and dose-response meta-analysis

# Supplementary materials

**Authors**

Diyang Lyu[1#], Rui Zhuang[1#], Jiaqi Li[1#], Yucen Wu[2], Yiming Di[3], Meifen Song[3], Liyong Ma[1], Jingen Li[1*], Yong Zhang[4, 5*].

# Content

Table S1. Adding characteristics of the included studies. ................................................................4
Table S2. Subgroup analysis of the association between hyperuricemia and risk of coronary heart disease and other cardiovascular outcomes by different statistics. ................................................25
Table S3. Subgroup analysis of the association between increasing unit of serum uric acid and risk of coronary heart disease and other cardiovascular outcomes by different statistics. .....................26
Table S4. Meta-regression of the association between hyperuricemia and risk of coronary heart disease and other cardiovascular outcomes by measuring age, sex, and BMI. ...............................27
Table S5. Meta-regression of the association between increasing unit serum UA and risk of coronary heart disease and other cardiovascular outcomes by measuring age and sex.................................28
Supplementary Figures S1. Traffic light plot of quality assessment of each included study. .........29
Supplementary Figures S2. Forest plots, sensitivity analysis, and funnel plots of association between hyperuricemia and cardiovascular outcomes, with or without the inclusion of quantile data.........30
Supplementary Figures S3. Forest plots, sensitivity analysis, and funnel plots of association between increase of 1 unit or 1mg/dL of serum UA and cardiovascular outcomes......................................45
Supplementary Figures S4. Restricted cubic spline regression plots of dose-response meta-analysis. ..........................................................................................................................................59

## Abbreviations

| | |
|---|---|
| F | Female |
| M | Male |
| CVD | Cardiovascular disease |
| CHD | Coronary |
| MI | Myocardial infarction |

**Table S1. Adding characteristics of the included studies.**

| Study | Original study initiation year | Age range | BMI (Mean, SD) | Whole study sample size | Outcome | Outcome definition | Outcome determination | Type of statistics | Confounding factors | Number of confounding factors | Grouping method based on uric acid | Attending rate |
|---|---|---|---|---|---|---|---|---|---|---|---|---|
| Fessel 1980 | 1968 | 20-49 | Not mentioned. | 325 | CHD | Typical angina, MI, intermittent claudication, cerebral thrombosis or hemorrhage | Based on medical records. | Not mentioned. | None. | 0 | ≥ 2 SD from mean level. | 93.50% |
| Goldberg 1995 | 1965 | 55-64 | 23.5 | 8006 | CHD | Fatal CHD and non-fatal MI. | Researchers determined based on medical records and family members' reports. | RR | Ventricular rate, BMI, SBP, T-CHO, TG, glucose, hematocrit, forced expiratory volume, physical activity, cigarettes per day, alcohol consumption. | 12 | Not mentioned | 33.80% |
| Wannamethee 1997 | 1978 | 40-59 | Categorized data | 7735 | CHD | Non-fatal MI and fatal CHD (ICD-9 410-414). | Death: official records with ICD- | Not mentioned. | Age, smoking, alcohol | 5 | Not mentioned | 99.00% |

| Study | Original study initiation year | Age range | BMI (Mean, SD) | Whole study sample size | Outcome | Outcome definition | Outcome determination | Type of statistics | Confounding factors | Number of confounding factors | Grouping method based on uric acid | Attending rate |
|---|---|---|---|---|---|---|---|---|---|---|---|---|
| | | | | | | | 9 code; CHD: physican report or regular review of notes. | | intake, BMI, diabetes. | | | |
| Culleton 1999 | 1971 | 35-74 | 25.66, 4.34 | 6763 | CHD and CVD death. | CHD: death from CHD, MI, and coronary insufficiency. Angina prctoris were not included. | Researchers determined based on medical records. | HR | Age, BMI, SBP, use of antihypertensive agents, use of diuretics, diabetes, T-CHO, smoking status, alcohol intake, LVH, and menopausal status. | 11 | Not mentioned | 96.30% |
| Liese 1999 | 1984 | 25-64 | 27.70, 3.29 | 5069 | CVD death and MI. | CHD: nonfatal AMI and fatal CHD (ICD-9 390-459). | Death: official records and researcher's | HR | Age, alcohol, TC/HDL-C ratio, hypertention, diuretic drug use, smoking, | 8 | Not mentioned | 99.70% |

| Study | Original study initiation year | Age range | BMI (Mean, SD) | Whole study sample size | Outcome | Outcome definition | Outcome determination | Type of statistics | Confounding factors | Number of confounding factors | Grouping method based on uric acid | Attending rate |
|---|---|---|---|---|---|---|---|---|---|---|---|---|
| | | | | | | | determination with ICD-9 code; for CHD, with WHO MONICA criteria. | | BMI, education. | | | |
| Fang 2000 | 1971 | 25-74 | 25.73, 5.14 | 20729 | Death for cardiovascular disease and ischemic heart disease. | Total cardiovascular disease (ICD-9 390-459), ischemic heart disease (ICD-9 410-414). | Based on medical records with ICD-9 codes, death certificates, and interviews. | RR | age, sex, T-CHO, race, BMI, smoking status, al-cohol consumption, and history of hypertension and diabetes. | 8 | Not mentioned | 96.40% |
| Moriarity 2000 | 1987 | 45-64 | 27.65 | 15792 | CHD | A validated definite or probable MI or definite CHD death. | Based on medical records. | RR | Age, race, study field center, smoking status, cigarette-years, SBP, hypertensive | 16 | Not mentioned | 80.00% |

| Study | Original study initiation year | Age range | BMI (Mean, SD) | Whole study sample size | Outcome | Outcome definition | Outcome determination | Type of statistics | Confounding factors | Number of confounding factors | Grouping method based on uric acid | Attending rate |
|---|---|---|---|---|---|---|---|---|---|---|---|---|
| | | | | | | | | | medication use, LDL-C, alcohol, protein intake, TG, HDL-C, BMI, waist/hip ratio, sports index, and diabetes. | | | |
| Jee 2004 | 1992 | 30-77 | 23.8, 2.8 | 22698 | ASCVD death | IHD death and stroke death | Official ICD-9 and ICD-10 records. | RR | Age, smoking, BMI, T-CHO, alcohol drinking, exercise and diabetes. | 7 | Not mentioned | 100.00% |
| Baibas 2005 | 1981 | Not mentioned. | Not mentioned. | 1198 | CHD death | ICD-9 codes 410-414 and all cases of sudden cardiac death: code 427.5. | Official ICD-9 code and ascertained by a researcher. | HR | Age, village, education, body weight, smoking, alcohol, SBP, blood glucose, TG. | 9 | Not mentioned. | 95.99% |

| Study | Original study initiation year | Age range | BMI (Mean, SD) | Whole study sample size | Outcome | Outcome definition | Outcome determination | Type of statistics | Confounding factors | Number of confounding factors | Grouping method based on uric acid | Attending rate |
|---|---|---|---|---|---|---|---|---|---|---|---|---|
| Chien 2005 | 1990 | >35 | 23.84, 4.79 | 4349 | CHD | Coronary death, non-fatal MI, and hospitalization due to procedures of CABG and PCI. Slient MIs were not included. | Researchers determined with interviews and death certificates. | HR | Age, SBP, BMI, diabetes, LDL-C, HDL-C, smoking and drinking status. | 8 | ⩾7.7mg/dl in men or ⩾ 6.6mg/dl in women. | 50.24% |
| Bos 2006 | 1990 | ⩾55 | Not mentioned. | 7983 | CHD | MI, a revascularization procedure (percutaneous transluminal coronary angioplasty or CABG), other forms of acute or chronic ischemic heart disease, sudden (cardiac) death, and death attributable to ventricular fibrillation and congestive heart failure. | Researchers determined and coded with ICD-10. | HR | Age, sex, SBP, T-CHO, HDL-C, diatebes, smoking, diuretic use, waist/hip ratio. | 9 | Not mentioned | 97.10% |

| Study | Original study initiation year | Age range | BMI (Mean, SD) | Whole study sample size | Outcome | Outcome definition | Outcome determination | Type of statistics | Confounding factors | Number of confounding factors | Grouping method based on uric acid | Attending rate |
|---|---|---|---|---|---|---|---|---|---|---|---|---|
| Gerber 2006 | 1963 | ⩾40 | 25.61, 3.35 | 10059 | CHD death | ICD-9 codes 410 to 414. | Researchers reviewed medical records and death certificates, and coded with ICD-9. | HR | Age, BMI, SBP, diabetes, T-CHO, smoking, and LVH on electrocardiogram. | 7 | Not mentioned | 98.00% |
| Iwashima 2006 | Not mentioned. | Not mentioned. | 24.39, 0.28 | 619 | CVD events. | MI, AP, stroke, transient cerebral ischemia and CHF. | Researchers determined with clinical symptoms and auxiliary examinations. | HR | Age, LVMI, sex, BMI, duration of hypertention, smoking, SBP, DBP, pulse pressure, heart rate, diabetes, T-CHO, TG, HDL-C, Ccr, HOMA, CRP. | 17 | Median value of the study (6.25mg/dl in men and 5.06mg/dl in women). | Not mentioned. |

| Study | Original study initiation year | Age range | BMI (Mean, SD) | Whole study sample size | Outcome | Outcome definition | Outcome determination | Type of statistics | Confounding factors | Number of confounding factors | Grouping method based on uric acid | Attending rate |
|---|---|---|---|---|---|---|---|---|---|---|---|---|
| Baba 2007 | 1990 | Not mentioned. | 22.73, 3.20 | 7564 | CHD | MI and AP. | Researchers determined with interviews and medical records. | RR | Age, sex, smoking, alcohol, glucose intolerance, and fatty liver. | 7 | ≥7.0mg/dl. | Not mentioned. |
| Strasak 2008a | 1985 | Not mentioned. | 25.3, 3.6 | 85035 | CHD death | Acute and subacute forms of CHD, chronic forms of CHD including occlusive CHD and its complications, hemorrhagic stroke, ischemic stroke, undefined stroke. | Confirmed with death certificates and recoded with ICD-9 | HR | Age, BMI, DBP, SBP, T-CHO, TG, GGT, blood glucose, smoking status, and year of examination. | 10 | Not mentioned. | Not mentioned. |
| Strasak 2008b | 1985 | >50 | 26.5, 4.6 | 28613 | CVD death and CHD death | Acute and subacute forms of CHD, chronic forms of CHD including occlusive CHD and its complications, hemorrhagic stroke, ischemic stroke, undefined stroke. | Confirmed with death certificates and recoded with ICD-9 | HR | Age, BMI, DBP, SBP, T-CHO, TG, GGT, blood glucose, smoking status, and year of examination. | 10 | Not mentioned. | Not mentioned. |

| Study | Original study initiation year | Age range | BMI (Mean, SD) | Whole study sample size | Outcome | Outcome definition | Outcome determination | Type of statistics | Confounding factors | Number of confounding factors | Grouping method based on uric acid | Attending rate |
|---|---|---|---|---|---|---|---|---|---|---|---|---|
| Chen 2009 | 1994 | ⩾35 | 24.20, 8.91 | 146900 | CVD death and CHD death | Death due to total CVD, ischemic stroke, hemorrhagic stroke, coronary heart disease, CHF, hypertensive disease. | Official ICD-9 code | HR | Age, sex, BMI, T-CHO, TG, diabetes, hypertension, heavy cigarette smoking, and frequent alcohol consumption. | 9 | >7.0mg/dl. | Not mentioned. |
| Holme 2009 | 1985 | | Not mentioned. | 417734 | AMI | ICD 8 and ICD 9: 410, ICD 10: I21. | Official ICD-7 to ICD-10 codes | HR | Age, TC, TG, hypertension and diabetes. | 5 | Not mentioned. | Not mentioned. |
| Chuang 2012 | 1994 | >20 | 23.33, 6.56 | 128569 | IHD | ICD-9-CM codes 410-414. | Official ICD-9 code | HR | Age, SBP, DBP, drugs using for hypertension, diuretics using during follow-up period, BMI, TG, T-CHO, diabetes, smoking, alcohol | 13 | ⩾7.0mg/dl in men or ⩾ 6.0mg/dl in women. | Not mentioned. |

| Study | Original study initiation year | Age range | BMI (Mean, SD) | Whole study sample size | Outcome | Outcome definition | Outcome determination | Type of statistics | Confounding factors | Number of confounding factors | Grouping method based on uric acid | Attending rate |
|---|---|---|---|---|---|---|---|---|---|---|---|---|
| | | | | | | | | | drinking, physical activity, and working type. | | | |
| Kawai 2012 | 1998 | Not mentioned. | 24.2, 0.1 | 774 | CVD | AP, STEMI, heart failure, and rupture of an aortic aneurysm. | Researchers determined with clinical symptoms and auxiliary examinations. | RR | Sex, BMI, DBP, TG, HDL-C, creatinine, HbA1C. | 7 | Not mentioned. | 93.50% |
| Kivity 2013 | 2001 | >34 | 26.06, 3.73 | 18034 | CVD | CHD, MI, cerebral vascular events, or transient ischemic attacks. | Researchers determined with interviews. | HR | Age, serum creatinine level, BMI, SBP, LDL-C, TG, plasma fasting glucose, physical activity, cardiovascular family history, use of diuretics, | 11 | ⩾7.2mg/dl in men or ⩾6.0mg/dl in women. | Not mentioned. |

| Study | Original study initiation year | Age range | BMI (Mean, SD) | Whole study sample size | Outcome | Outcome definition | Outcome determination | Type of statistics | Confounding factors | Number of confounding factors | Grouping method based on uric acid | Attending rate |
|---|---|---|---|---|---|---|---|---|---|---|---|---|
| | | | | | | | | | and current smoking. | | | |
| Onat 2013 | 2003 | Not mentioned. | 27.55, 4.71 | 1693 | CHD | CHD death: death from heart failure of coronary origin and fatal coronary event. Nonfatal CHD: AP, a history of MI or myocardial revascularization. | For death: family reports or official records. For CHD: Researchers determined with clinical symptoms and auxiliary examinations. | HR | Sex, age, alcohol usage, physical activity, systolic BP, HDL-C. | 6 | Not mentioned. | 89.07% |
| Shiozaki 2013 | 1996 | 34-60 | 24.57 | 174 | CHD | MI, AP, and ACS. | Researchers determined with medical records. | OR | Age, fat, hypertention, hypertriglyceridemia, hyperlipidemia, low HDL-C, impaired glucose | 9 | ⩾7.0mg/dl in men or ⩾ 6.0mg/dl in women. | 100.00% |

| Study | Original study initiation year | Age range | BMI (Mean, SD) | Whole study sample size | Outcome | Outcome definition | Outcome determination | Type of statistics | Confounding factors | Number of confounding factors | Grouping method based on uric acid | Attending rate |
|---|---|---|---|---|---|---|---|---|---|---|---|---|
| | | | | | | | | | tolerance, hyperuricemia, smoking. | | | |
| Storhaug 2013 | 1994 | 55-75 | 25.93, 3.28 | 27158 | MI | First fatal or nonfatal MI | Researchers determined with medical records and death certificates. | HR | Age, BMI, SBP, DBP, HDL-C, T-CHO, use of diuretics and other antihypertensive medication, current smoking and physical activity, current smoking, GFR, and log ACR. | 11 | Not mentioned. | 75.00% |
| Puddu 2014 | 1983 | 35-74 | 27.41, 1.04 | 5382 | CVD, CHD, CVD death and CHD death | SCD, MI, stroke, surgery of carotid arteries, aortic aneurism, fatal lower limbs artery disease, surgery of aorta or lower limb | Official ICD-9 code | HR | Age, gender, SBP,T-CHO, HDL-C, cigarette consumption, BMI, blood glucose, | 9 | Not mentioned. | 92.20% |

| Study | Original study initiation year | Age range | BMI (Mean, SD) | Whole study sample size | Outcome | Outcome definition | Outcome determination | Type of statistics | Confounding factors | Number of confounding factors | Grouping method based on uric acid | Attending rate |
|---|---|---|---|---|---|---|---|---|---|---|---|---|
| | | | | | | arteries, heart failure or chronic arrhythmia of uncertain etiology and leading to death, and fatal chronic coronary heart disease. | | | eGFR_CKDEPI. | | | |
| Wang 2015 | 1985 | 18-30 | 24.5, 5.0 | 5115 | Fatal and non-fatal CVD. | MI, AP, death due to CHD, stroke, transient ischemic attack, heart failure, and peripheral artery disease. | Researchers determined with medical records. | HR | Sex, race, age, clinic, education, total energy, alcohol, protein intakes at the year of sUA measurement, lifestyle factors, BMI, metabolic syndrome, insulin resistance, CHO/HDL-C, blood pressure, waist | 18 | ⩾6.8mg/dl | 95.00% |

| Study | Original study initiation year | Age range | BMI (Mean, SD) | Whole study sample size | Outcome | Outcome definition | Outcome determination | Type of statistics | Confounding factors | Number of confounding factors | Grouping method based on uric acid | Attending rate |
|---|---|---|---|---|---|---|---|---|---|---|---|---|
| | | | | | | | | | circumference, usage of anti-hypertension medication, the usage of diuretics, and glomerular filtration rate. | | | |
| Lai 2016 | 2008 | Not mentioned. | 24.38, 3.37 | 27009 | CHD | AP, AMI, subsequent MI, other forms of acute or chronic heart disease, percutaneous transluminal coronary angioplasty or CABG, and cardiac arrest or death with CHD. | Official ICD-9 code | HR | age, gender, BMI, smoking status, drinking status, physical activity, education levels, hypertension, diabetes, hyperlipidemia, a family history of CHD, use of diuretics, eGFR, and | 14 | ⩾7.0mg/dl in men or ⩾ 6.0mg/dl in women. | 96.20% |

| Study | Original study initiation year | Age range | BMI (Mean, SD) | Whole study sample size | Outcome | Outcome definition | Outcome determination | Type of statistics | Confounding factors | Number of confounding factors | Grouping method based on uric acid | Attending rate |
|---|---|---|---|---|---|---|---|---|---|---|---|---|
| | | | | | | | | | diet frequency categories. | | | |
| Zhang 2016 | 1977 | 35-89 | 22.88 | 36313 | CVD death | Ischemic stroke, hemorrhagic stroke, CHD, and heart failure. | Official ICD-9 code | HR | Age, smoking, alcohol, BMI, TG, T-CHO, SBP. | 7 | Not mentioned. | Not mentioned. |
| Wu 2017 | 2009 | ⩾65 | 22.46, 1.60 | 2142 | CAD events | MI and coronary insufficiency. Events that were more equivocal, such as unrecognized myocardial infarction and angina pectoris, were not included. | Researchers determined with medical records and family reports. | HR | Age, sex, smoking and drinking habits, baseline BMI, baseline SBP and DBP, and baseline eGFR, T-CHO, TG, LDL and HDL. | 11 | ⩾7.0mg/dl in men or ⩾ 6.0mg/dl in women. | 100.00% |
| Andrikou 2018 | Not mentioned. | Not mentioned. | 28.20, 4.09 | 2287 | CAD events | CAD | Researchers determined with medical records. | HR | Age, sex, waist circumference, diabetes, CKD, LVMI, LVH. | 7 | 5.2mg/dl | Not mentioned. |

| Study | Original study initiation year | Age range | BMI (Mean, SD) | Whole study sample size | Outcome | Outcome definition | Outcome determination | Type of statistics | Confounding factors | Number of confounding factors | Grouping method based on uric acid | Attending rate |
|---|---|---|---|---|---|---|---|---|---|---|---|---|
| Boutet 2020 | Not mentioned. | 40-70 | Not mentioned. | Not mentioned. | CVE and CVI | CVE: ACS, HF, strokes, CV deaths and peripheral vascular events. CVI: coronary, carotid, or peripheral artery revascularization. | Not mentioned. | HR | Age, smoking, family history of MI, hypertension, uric acid, GGT, waist-hip ratio, glycosylated hemoglobin, and prior cancer. | 9 | Not mentioned. | Not mentioned. |
| Tian 2020 | 2006 | 18-98 | 25.1, 3.5 | 101510 | MIs | MI | Researchers determined with medical records. | HR | Age, sex, education, current smoker, current alcohol, physical activity, BMI, FBG, SBP, DBP, hypertension, diabetes, dyslipidemia, antihypertensive | 18 | ⩾7.0mg/dl in men or ⩾ 6.0mg/dl in women. | Not mentioned. |

| Study | Original study initiation year | Age range | BMI (Mean, SD) | Whole study sample size | Outcome | Outcome definition | Outcome determination | Type of statistics | Confounding factors | Number of confounding factors | Grouping method based on uric acid | Attending rate |
|---|---|---|---|---|---|---|---|---|---|---|---|---|
| | | | | | | | | | medication, antidiabetic medication, lipid-lowering medication at baseline, eGFR and CRP. | | | |
| Cheng 2021 | 2011 | 18-91 | Categorized data | 45674 | CVD | First-time CHD and stroke attack cases. CHD: HF, MI, coronary angioplasty implant and graf. Stroke: ischemic stroke, hemorrhagic stroke, and hypertensive heart disease. | Official ICD-10 code | HR | Age at diagnosis, sex, BMI, smoking index, lifetime total alcohol intake, diabetes status, hypertension stage, TG, HDL-C, and LDL-C. | 12 | ⩾6.0mg/dl | 81.10% |

| Study | Original study initiation year | Age range | BMI (Mean, SD) | Whole study sample size | Outcome | Outcome definition | Outcome determination | Type of statistics | Confounding factors | Number of confounding factors | Grouping method based on uric acid | Attending rate |
|---|---|---|---|---|---|---|---|---|---|---|---|---|
| Colantonio 2021 | 2003 | >45 | Categorized | 3151 | SCD and CHD | CHD: MI or CHD death, exclude SCD | Researchers determined with medical records. | HR | Age, sex, race, geographic region of residence, income, education, alcohol consumption, current smoking, BMI, physical activity, CKD, history of stroke, diabetes, atrial fibrillation, SBP, T-CHO, HDL-C, CRP, LVH, SLC2A9 single-nucleotide polymorphisms, and use | 24 | ⩾6.8mg/dl | 94.60% |

| Study | Original study initiation year | Age range | BMI (Mean, SD) | Whole study sample size | Outcome | Outcome definition | Outcome determination | Type of statistics | Confounding factors | Number of confounding factors | Grouping method based on uric acid | Attending rate |
|---|---|---|---|---|---|---|---|---|---|---|---|---|
| | | | | | | | | | of antihypertensive medication, diuretics, statin, and allopurinol. | | | |
| Podpalov 2022 | 2007 | Not mentioned. | Not mentioned. | 3500 | MI and CVD death | Not mentioned. | Not mentioned. | Not mentioned. | Age, sex, HF, hypertention, edema, low HDL-C, COPD. | 7 | ⩾5.68mg/dl | 84.30% |
| Lee 2023 | 2006 | ⩾20 | 23.2, 3.0 | 20530 | IHD | Angina or AMI. | Official ICD-10 code | HR | Age, sex, BMI, smoking status, alcohol intake, physical activity, mean arterial blood pressure, fasting plasma glucose, T-CHO, hypertension | 11 | ⩾6.5mg/dl in men or ⩾ 4.6mg/dl in women. | Not mentioned. |

| Study | Original study initiation year | Age range | BMI (Mean, SD) | Whole study sample size | Outcome | Outcome definition | Outcome determination | Type of statistics | Confounding factors | Number of confounding factors | Grouping method based on uric acid | Attending rate |
|---|---|---|---|---|---|---|---|---|---|---|---|---|
| | | | | | | | | | medication, and dyslipidemia medication. | | | |
| Perticone 2023 | 2001 | 22-72 | 26.9, 3.4 | 1650 | Coronary events, cerebrovascular events | Cerebrovascular events: MI, unstable AP, coronary revascularization, cardiovascular death or death for any cause, and stroke. | Researchers determined with medical records and death certificates. | HR | Age, BMI, smoking, T-CHO, HDL-C and LDL C, TG, SBP, HOMA and eGFR. | 9 | Not mentioned. | Not mentioned. |
| Tian 2023 | 2006 | 18-98 | 24.10, 3.29 | 101510 | CVD | Stroke and MI | Researchers determined with medical records. | HR | Age, sex, family history of CVD, categorical BMI, SBP, DBP, FBG, T-CHO, HDL-C, estimated glomerular filtration rate, and hs-CRP. | 11 | ⩾6.0mg/dl | Not mentioned. |

| Study | Original study initiation year | Age range | BMI (Mean, SD) | Whole study sample size | Outcome | Outcome definition | Outcome determination | Type of statistics | Confounding factors | Number of confounding factors | Grouping method based on uric acid | Attending rate |
|---|---|---|---|---|---|---|---|---|---|---|---|---|
| Wakabayashi 2023 | 2005 | Not mentioned. | 31.1, 5.9 | 450 | CVD and CHD | AP, MI, stroke, or arteriosclerosis obliterans. | Researchers determined with physican reports. | HR | Age, BMI, and anti-hyperuricemia medication. | 3 | ⩾7.0mg/dl | 74.44% |
| Boyarinova 2024 | 2012 | Not mentioned. | Not mentioned. | 4800 | CVD | MI, stroke, transient ischemic attack. | Not mentioned. | HR | BMI, hypertention, GFR, diabetes. | 4 | | 86.83% |
| Mayo-Juanatey 2025 | Not mentioned. | Not mentioned. | 26.7, 6.1 | 591 | CVE | MI, stroke, and peripheral arterial disease. | Not mentioned. | Not mentioned. | Not mentioned. | Not mentioned. | ⩾7.0mg/dl | Not mentioned. |
| Sarebanhassanabadi 2025 | 2005 | 20-74 | 26.16, 4.34 | 2000 | CAD | CAD, MI, PCI, CABG, and new-onset angina. | Researchers determined with medical records. | HR | Age, sex, smoking, physical activity, education, and family history, HDL-C, T-CHO, BMI, Waist to hip ratio, SBP, DBP, LDL-C. | 13 | Not mentioned. | 80.70% |

AMI, acute myocardial infarction; AP, angina pectoris; ASCVD, Atherosclerotic Cardiovascular Disease; BMI, body mass index; CABG, coronary artery bypass grafting; CAD, coronary artery disease; Ccr, creatinine clearance rate; CHD, coronary heart disease; CHF, chronic heart failure; CKDEPI, Chronic Kidney Disease Epidemiology Collaboration; COPD, chronic obstructive

pulmonary disease; Cox, Cox proportional hazards multivariate regression model; CRP, C-reactive protein; CVD, cardiovascular disease; CVE, cardiovascular event; CVI, cardiovascular intervention; DBP, diastolic blood pressure; eGFR, estimated Glomerular Filtration Rate; GGT, γ-Glutamyl transpeptidase; HbA1c, Hemoglobin A1c; HDL-C, high density lipoprotein cholesterol; HF, heart failure; HOMA, homeostatic model assessment index.; hs-CRP, high-sensitivity C-reactive protein; IHD, ischemic heart disease; LDL-C, low density lipoprotein cholesterol; LVH, left ventricular hypertrophy; LVMI, left ventricular mass index; MI, myocardial infarction; PCI, percutaneous coronary intervention; SBP, systolic blood pressure; SCD, suddent cardiac death; SD, standard deviation; T-CHO, total cholesterol; TG, triglyceride.

**Table S2. Subgroup analysis of the association between hyperuricemia and risk of coronary heart disease and other cardiovascular outcomes by different statistics.**

| Subgroup | Comparison | N of studies | HR (95%CI) | P value | $I^2$ | $Tau^2$ | P of Egger's test | Trim&Fill HR (95%CI) | P of Trim&Fill test | Trim&Fill difference |
|---|---|---|---|---|---|---|---|---|---|---|
| HR | | | | | | | | | | |
| | CHD | 10 | 1.21 (1.12, 1.3) | <0.001 | 41.39% | 0 | 0.438 | 1.18 (1.06, 1.32) | 0.003 | 0.024 |
| | CHD death | 5 | 1.15 (1.03, 1.27) | 0.011 | 0% | 0 | 0.097 | 1.1 (1, 1.21) | 0.055 | 0.394 |
| | CVD | 5 | 1.37 (1.18, 1.6) | <0.001 | 0% | 0 | 0.044 | 1.3 (1.14, 1.48) | <0.001 | 0.354 |
| | CVD death | 5 | 1.36 (1.25, 1.47) | <0.001 | 0% | 0 | 0.801 | 1.33 (1.24, 1.43) | <0.001 | 0.494 |
| | MI | 7 | 1.16 (1.08, 1.25) | <0.001 | 50.11% | 0 | 0.712 | 1.15 (1.07, 1.24) | <0.001 | 0.04 |
| RR | | | | | | | | | | |
| | CHD | 3 | 1.26 (0.95, 1.69) | 0.11 | 41.73% | 0.02 | 0.147 | 1.06 (0.75, 1.5) | 0.737 | 0.041 |
| | CHD death | 2 | 1.46 (0.62, 3.44) | 0.39 | 81.51% | 0.31 | | | | |

CHD, coronary heart disease; CI, confidence interval; CVD, cardiovascular disease; HR, hazard ratio; RR, risk ratio.

**Table S3. Subgroup analysis of the association between increasing unit of serum uric acid and risk of coronary heart disease and other cardiovascular outcomes by different statistics.**

| Subgroup | Comparison | N of studies | HR (95%CI) | P value | I2 | Tau2 | P of Egger's test | Trim&Fill HR (95%CI) | P of Trim&Fill test | Trim&Fill difference |
|---|---|---|---|---|---|---|---|---|---|---|
| HR | | | | | | | | | | |
| | CHD | 6 | 1.16 (1.05, 1.29) | 0.005 | 91.15% | 0.02 | 0.555 | 1.16 (1.05, 1.29) | 0.005 | <0.001 |
| | CHD death | 6 | 1.11 (1.08, 1.13) | <0.001 | 0% | 0 | 0.134 | 1.1 (1.08, 1.13) | <0.001 | 0.396 |
| | CVD | 5 | 1.1 (1.02, 1.19) | 0.01 | 85.49% | 0.01 | 0.037 | 1.02 (0.93, 1.13) | 0.616 | <0.001 |
| | CVD death | 3 | 1.11 (1.09, 1.12) | <0.001 | 0% | 0 | 0.757 | 1.11 (1.09, 1.12) | <0.001 | 0.888 |
| | MI | 5 | 1.07 (1.06, 1.09) | <0.001 | 0% | 0 | 0.182 | 1.07 (1.06, 1.09) | <0.001 | 0.46 |
| RR | | | | | | | | | | |
| | CHD death | 1 | 1.22 (1.14, 1.31) | <0.001 | | | | | | |
| | CVD | 1 | 1.36 (1.06, 1.74) | 0.015 | | | | | | |
| | CVD death | 1 | 1.17 (1.11, 1.24) | <0.001 | | | | | | |

CHD, coronary heart disease; CI, confidence interval; CVD, cardiovascular disease; HR, hazard ratio; MI, myocardial infarction; RR, risk ratio.

**Table S4. Meta-regression of the association between hyperuricemia and risk of coronary heart disease and other cardiovascular outcomes by measuring age, sex, and BMI.**

Meta-regression with age, sex, and BMI:

| | Age | | Sex | | BMI | |
|---|---|---|---|---|---|---|
| Outcome | β (95%CI) | P value | β (95%CI) | P value | β (95%CI) | P value |
| CHD death | 0 (-0.03, 0.04) | 0.851 | -0.91 (-1.66, -0.15) | 0.018 | 0.1 (-0.02, 0.23) | 0.095 |
| MI | 0 (-0.04, 0.04) | 0.977 | 0.01 (-0.01, 0.02) | 0.295 | 0.04 (-0.19, 0.28) | 0.705 |

Meta-regression with age and sex:

| | Age | | Sex | |
|---|---|---|---|---|
| Outcome | β (95%CI) | P value | β (95%CI) | P value |
| CHD | 0.01 (0, 0.02) | 0.061 | 0.7 (-0.48, 1.87) | 0.246 |
| CHD death | -0.02 (-0.07, 0.03) | 0.474 | -0.58 (-1.43, 0.27) | 0.179 |
| CVD | 0.01 (0, 0.03) | 0.187 | 0 (-0.01, 0) | 0.043 |
| CVD death | 0.02 (-0.02, 0.06) | 0.277 | 0.36 (-0.46, 1.18) | 0.392 |
| MI | -0.01 (-0.01, 0) | 0.206 | 0.01 (0, 0.01) | 0.111 |

BMI, body-mass index; CHD, coronary heart disease; CI, confidence interval; CVD, cardiovascular disease; MI, myocardial infarction.

**Table S5. Meta-regression of the association between increasing unit serum UA and risk of coronary heart disease and other cardiovascular outcomes by measuring age and sex.**

| | Age | | Sex | |
|---|---|---|---|---|
| Outcome | β (95%CI) | P value | β (95%CI) | P value |
| CHD | -0.01 (-0.02, 0.01) | 0.59 | -0.36 (-1.75, 1.03) | 0.612 |
| CHD death | -0.01 (-0.03, 0) | 0.068 | -0.26 (-0.57, 0.04) | 0.092 |
| CVD | 0 (-0.01, 0.01) | 0.611 | 0 (0, 0) | 0.872 |
| CVD death | -0.01 (-0.03, 0) | 0.097 | -0.27 (-0.64, 0.09) | 0.143 |
| MI | 0 (0, 0.01) | 0.57 | 0 (0, 0) | 0.181 |

CHD, coronary heart disease; CI, confidence interval; CVD, cardiovascular disease; MI, myocardial infarction.

## Supplementary Figures S1. Traffic light plot of quality assessment of each included study.

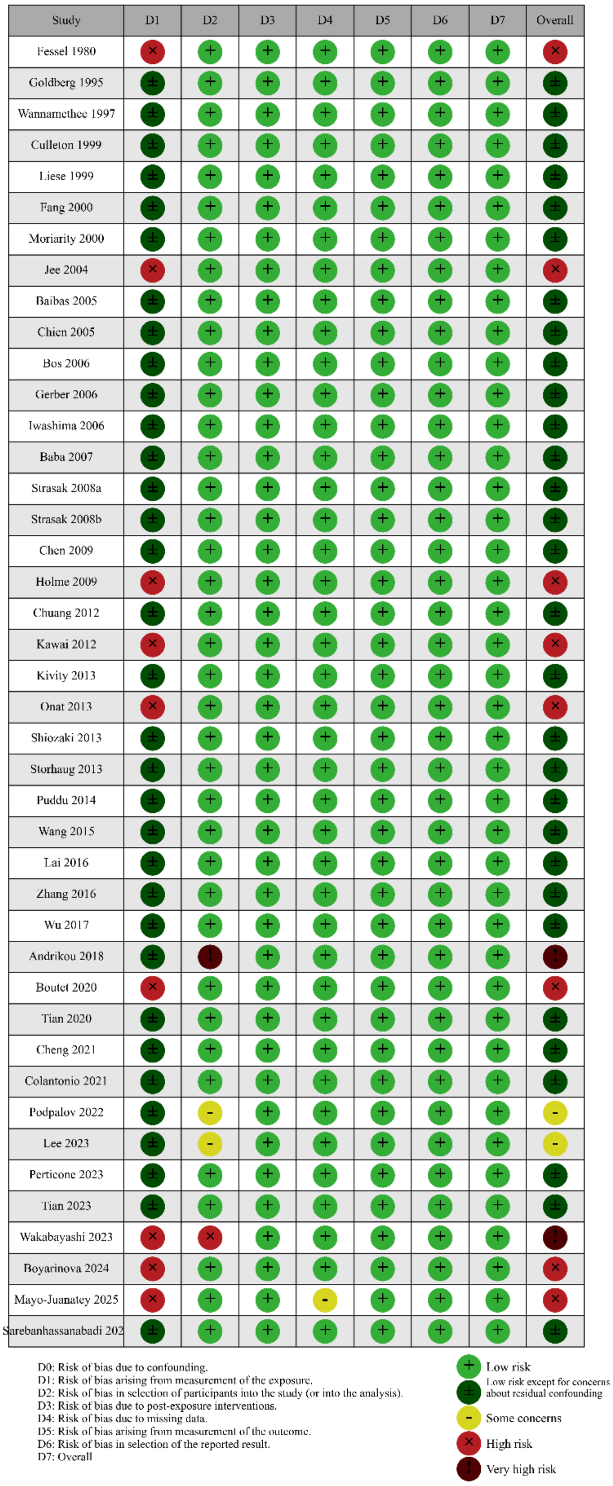


Figure S1. Traffic light plot of quality assessment of each included study.

**Supplementary Figures S2. Forest plots, sensitivity analysis, and funnel plots of association between hyperuricemia and cardiovascular outcomes, with or without the inclusion of quantile data.**

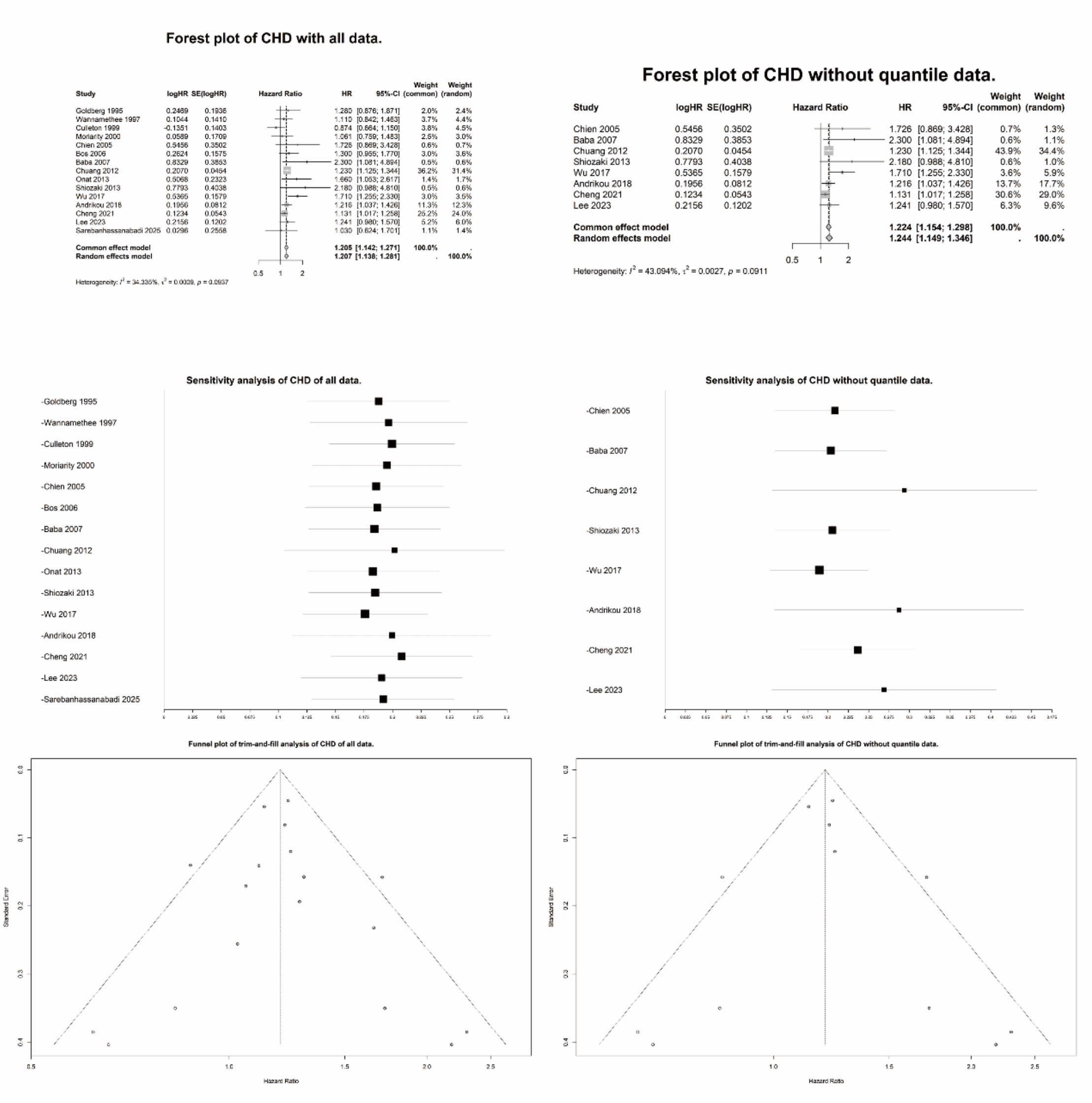


Figure S2-1. Forest plots, sensitivity analysis, and funnel plots of association between hyperuricemia and CHD, with or without the inclusion of quantile data. A total of 15 studies with 222,306 participants were included in the pooled estimation, while 8 studies with 186,264 participants were included after the exclusion of the studies with quantile data.

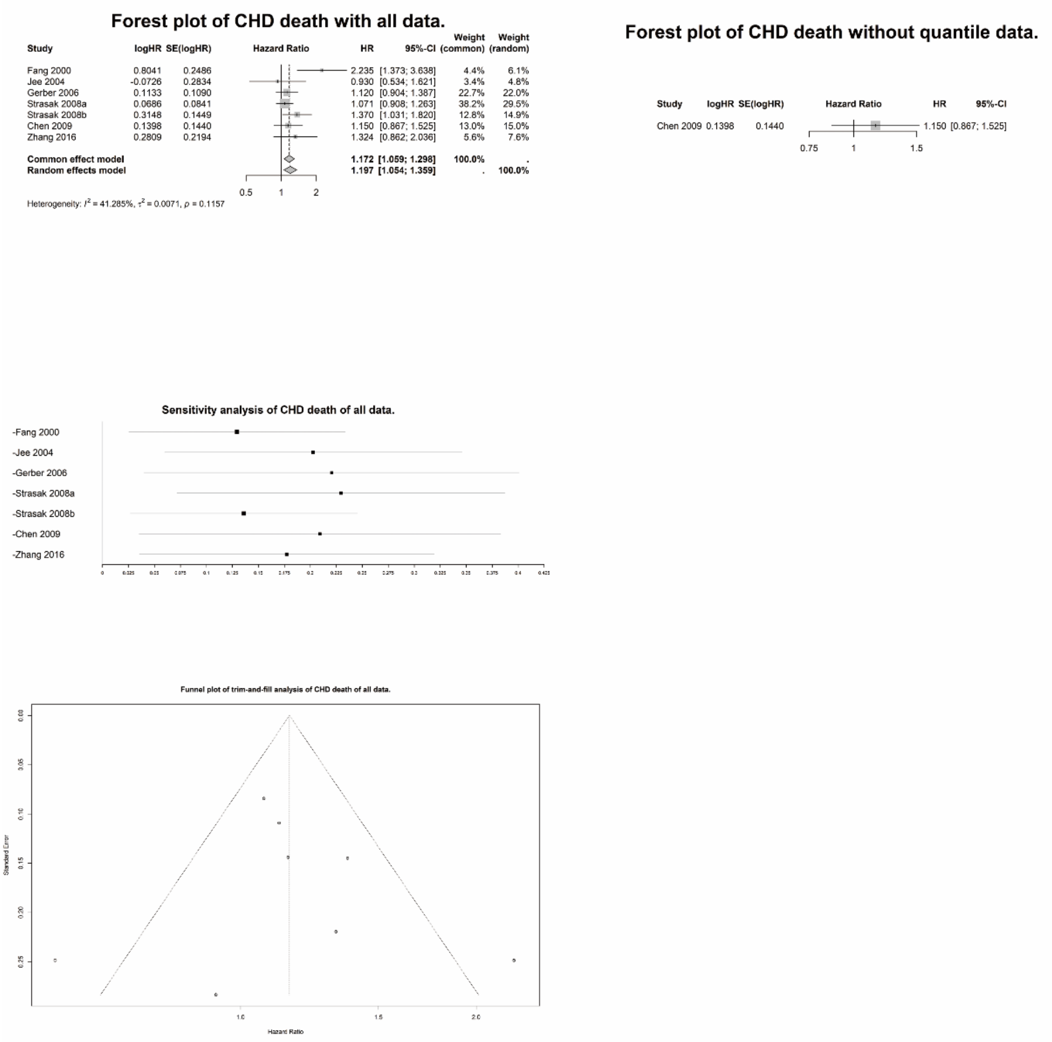


Figure S2-2. Forest plots, sensitivity analysis, and funnel plots of association between hyperuricemia and CHD death, with or without the inclusion of quantile data. A total of 7 studies with 276,751 participants were included in the pooled estimation, while 1 study with 90,393 participants were included after the exclusion of the studies with quantile data.

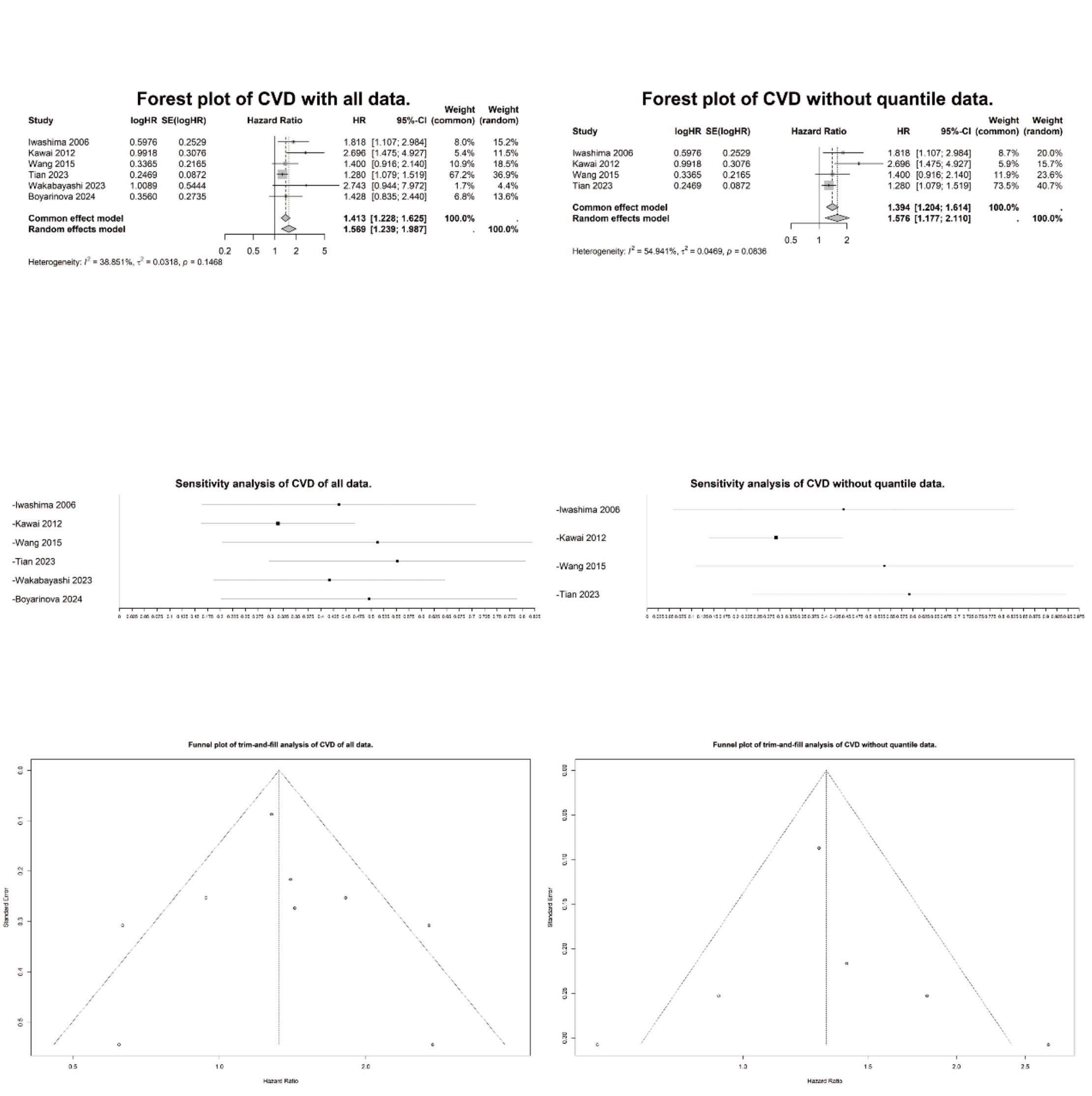


Figure S2-3. Forest plots, sensitivity analysis, and funnel plots of association between hyperuricemia and CVD, with or without the inclusion of quantile data. A total of 6 studies with 36,006participants were included in the pooled estimation, while 4 studies with 31,388 participants were included after the exclusion of the studies with quantile data.

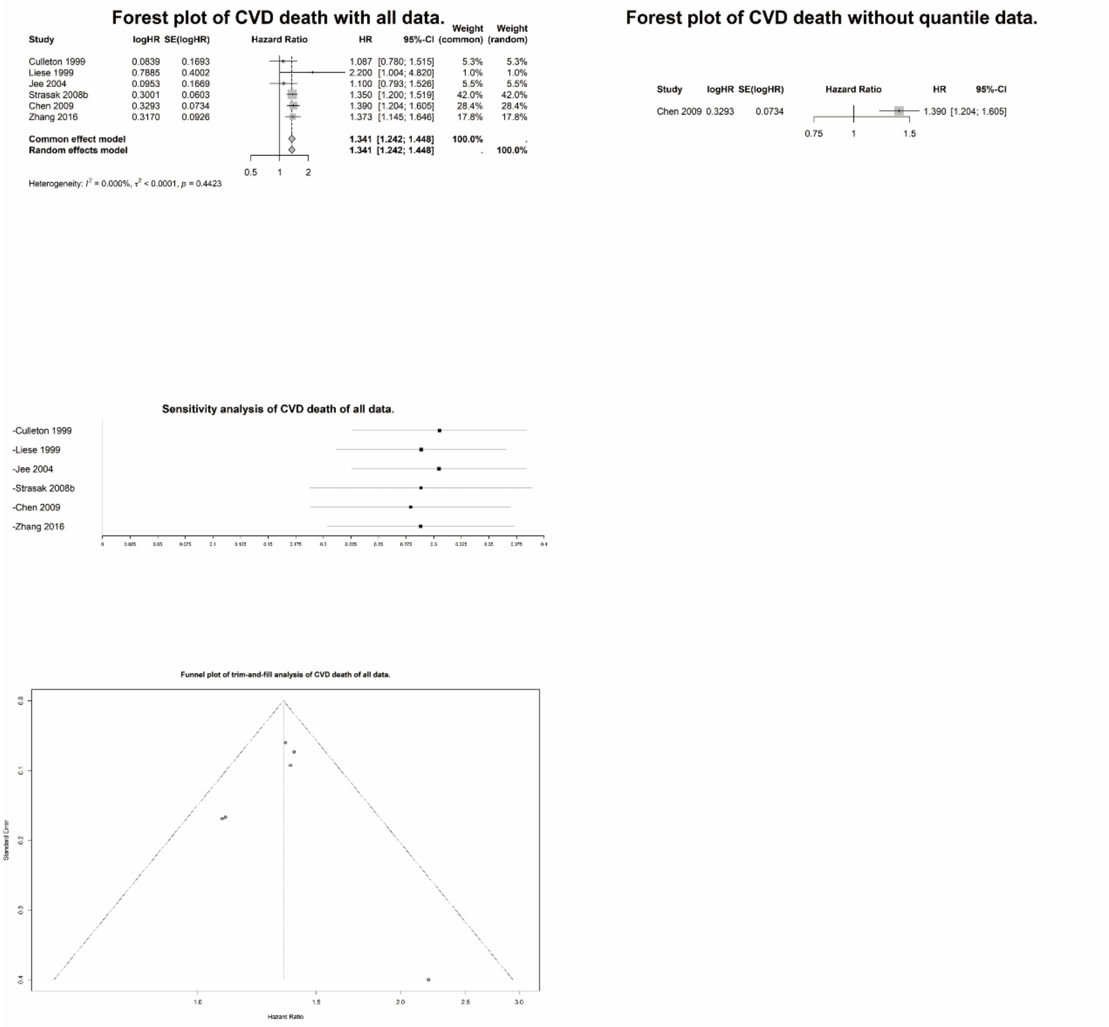


Figure S2-4. Forest plots, sensitivity analysis, and funnel plots of association between hyperuricemia and CVD death, with or without the inclusion of quantile data. A total of 6 studies with 185,854participants were included in the pooled estimation, while 1 study with 90,393 participants were included after the exclusion of the studies with quantile data.

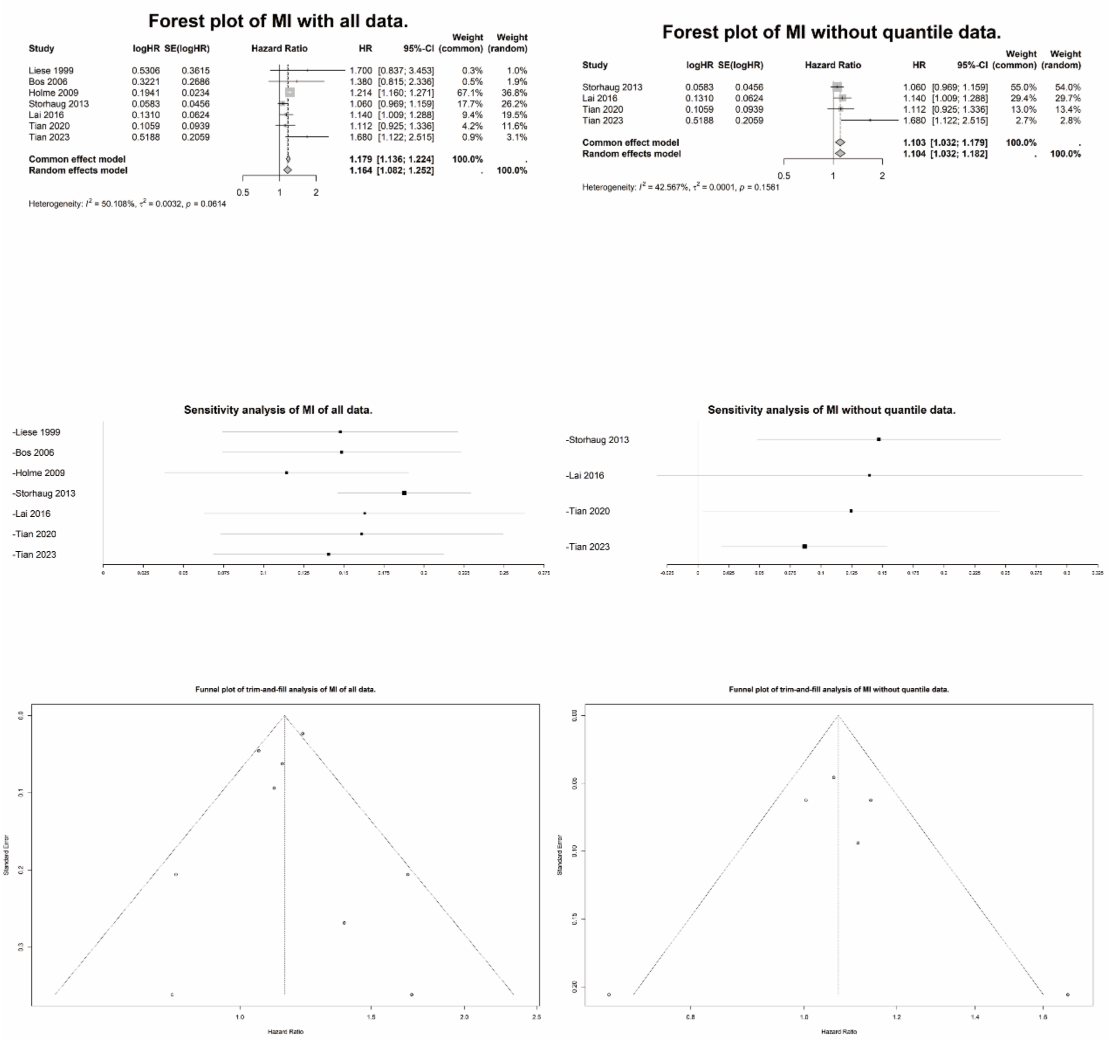


Figure S2-5. Forest plots, sensitivity analysis, and funnel plots of association between hyperuricemia and MI, with or without the inclusion of quantile data. A total of 7 studies with 541,689participants were included in the pooled estimation, while 4 studies with 118,496 participants were included after the exclusion of the studies with quantile data.

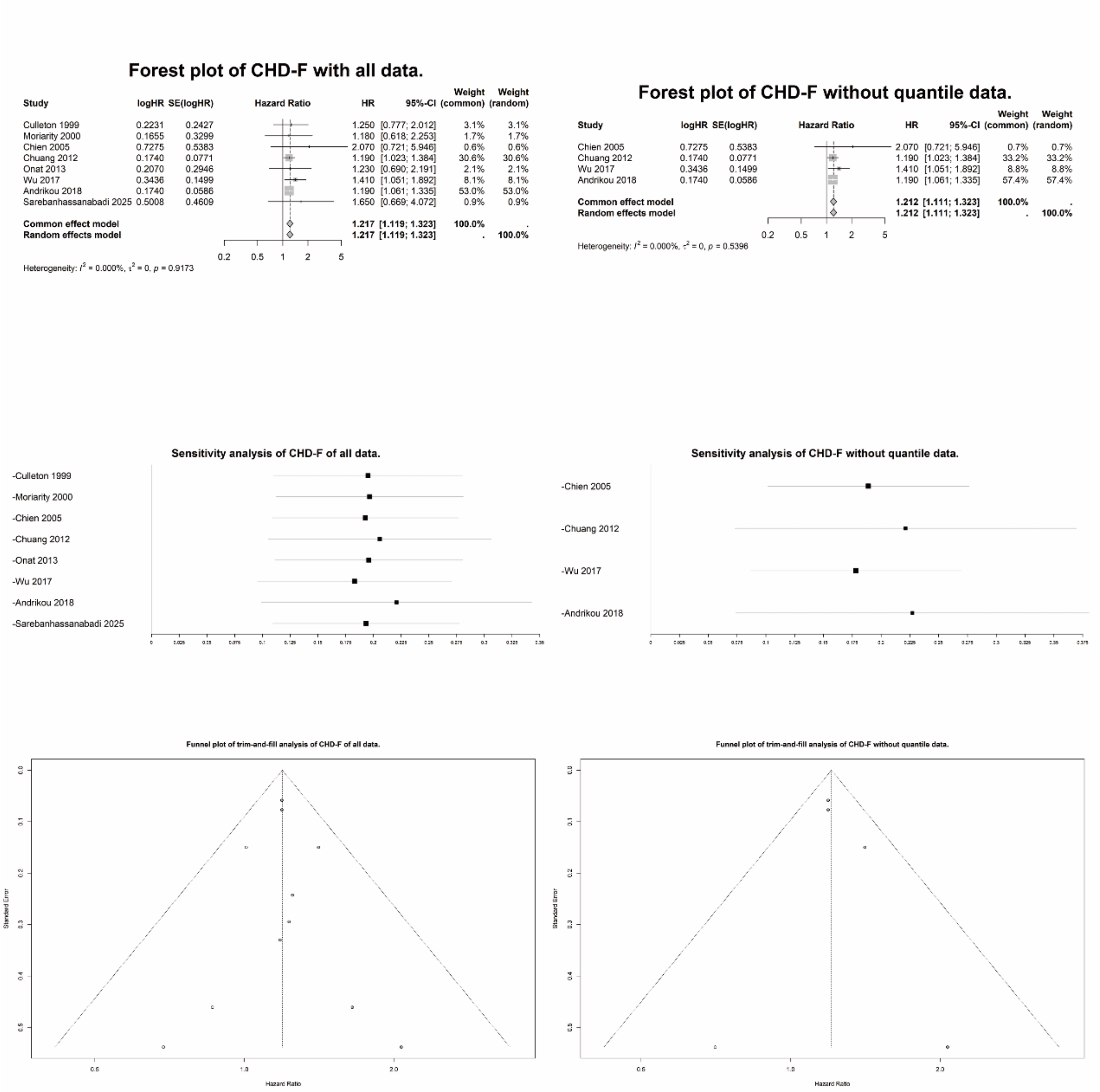


Figure S2-6. Forest plots, sensitivity analysis, and funnel plots of association between hyperuricemia and CHD among female population, with or without the inclusion of quantile data. A total of 8 studies with 85,432participants were included in the pooled estimation, while 4 studies with 72,678 participants were included after the exclusion of the studies with quantile data.

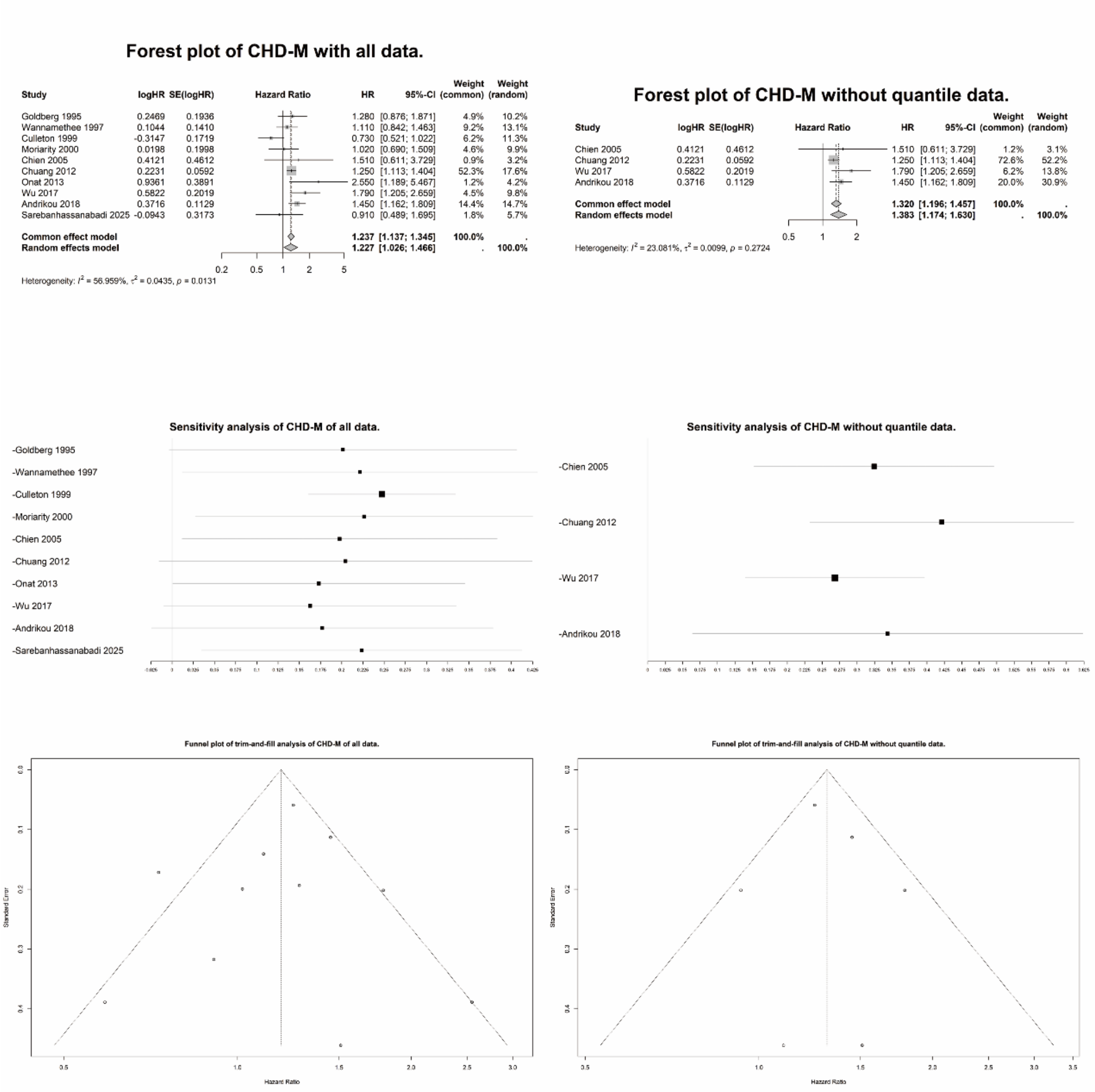


Figure S2-7. Forest plots, sensitivity analysis, and funnel plots of association between hyperuricemia and CHD among male population, with or without the inclusion of quantile data. A total of 10 studies with 82,825participants were included in the pooled estimation, while 4 studies with 63,922 participants were included after the exclusion of the studies with quantile data.

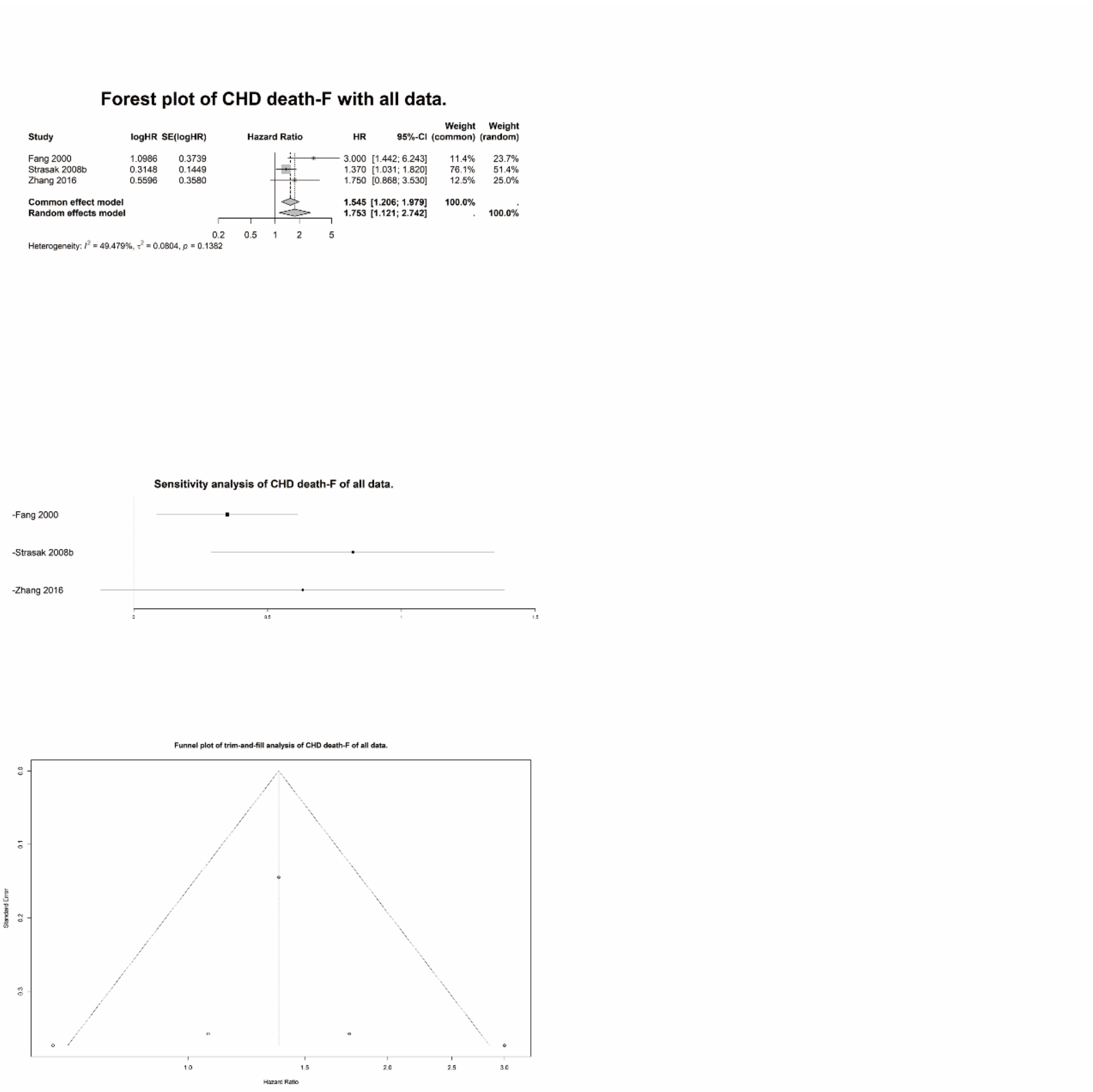


Figure S2-8. Forest plots, sensitivity analysis, and funnel plots of association between hyperuricemia and CHD death among female population, with the inclusion of quantile data. A total of 3 studies with 52,522 participants were included in the pooled estimation.

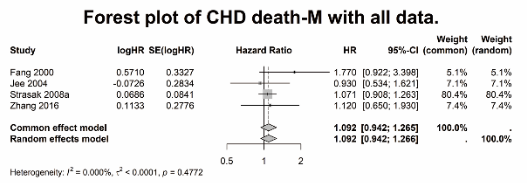


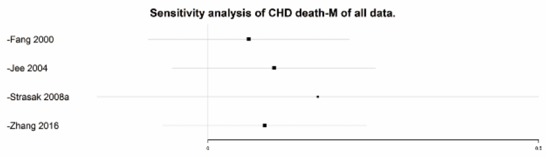


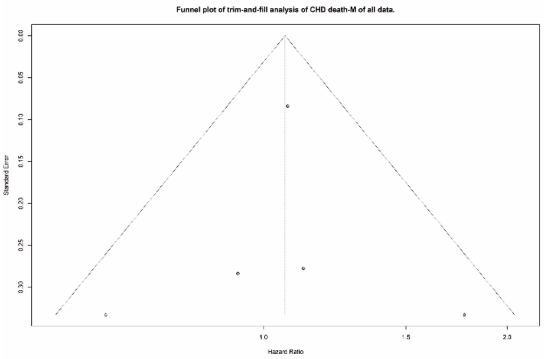


Figure S2-9. Forest plots, sensitivity analysis, and funnel plots of association between hyperuricemia and CHD death among male population, with the inclusion of quantile data. A total of 4 studies with 124,711 participants were included in the pooled estimation.

**Forest plot of CVD-F with all data.**

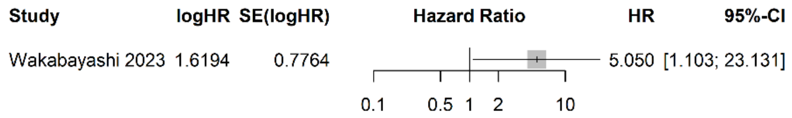


Figure S2-10. Forest plot of association between hyperuricemia and CVD among male population. A total of 1 study with 248 participants were included in the pooled estimation.

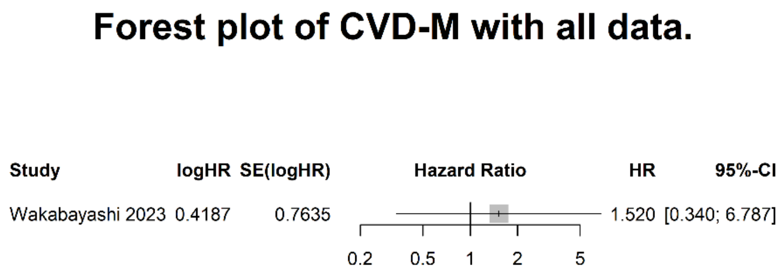


Figure S2-11. Forest plot of association between hyperuricemia and CVD among female population. A total of 1 study with 202 participants were included in the pooled estimation.

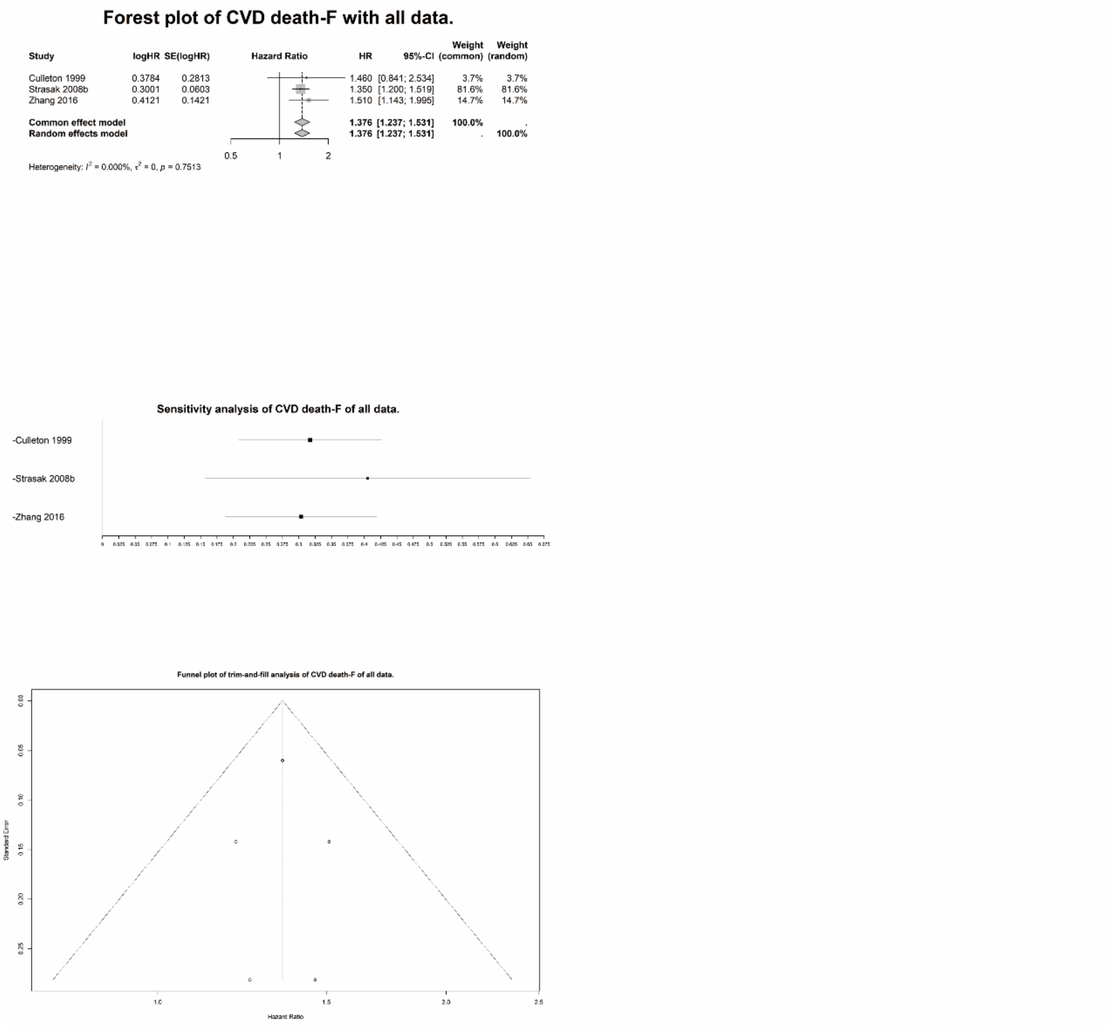


Figure S2-12. Forest plots, sensitivity analysis, and funnel plots of association between hyperuricemia and CVD death among female population, with the inclusion of quantile data. A total of 3 studies with 52,986 participants were included in the pooled estimation.

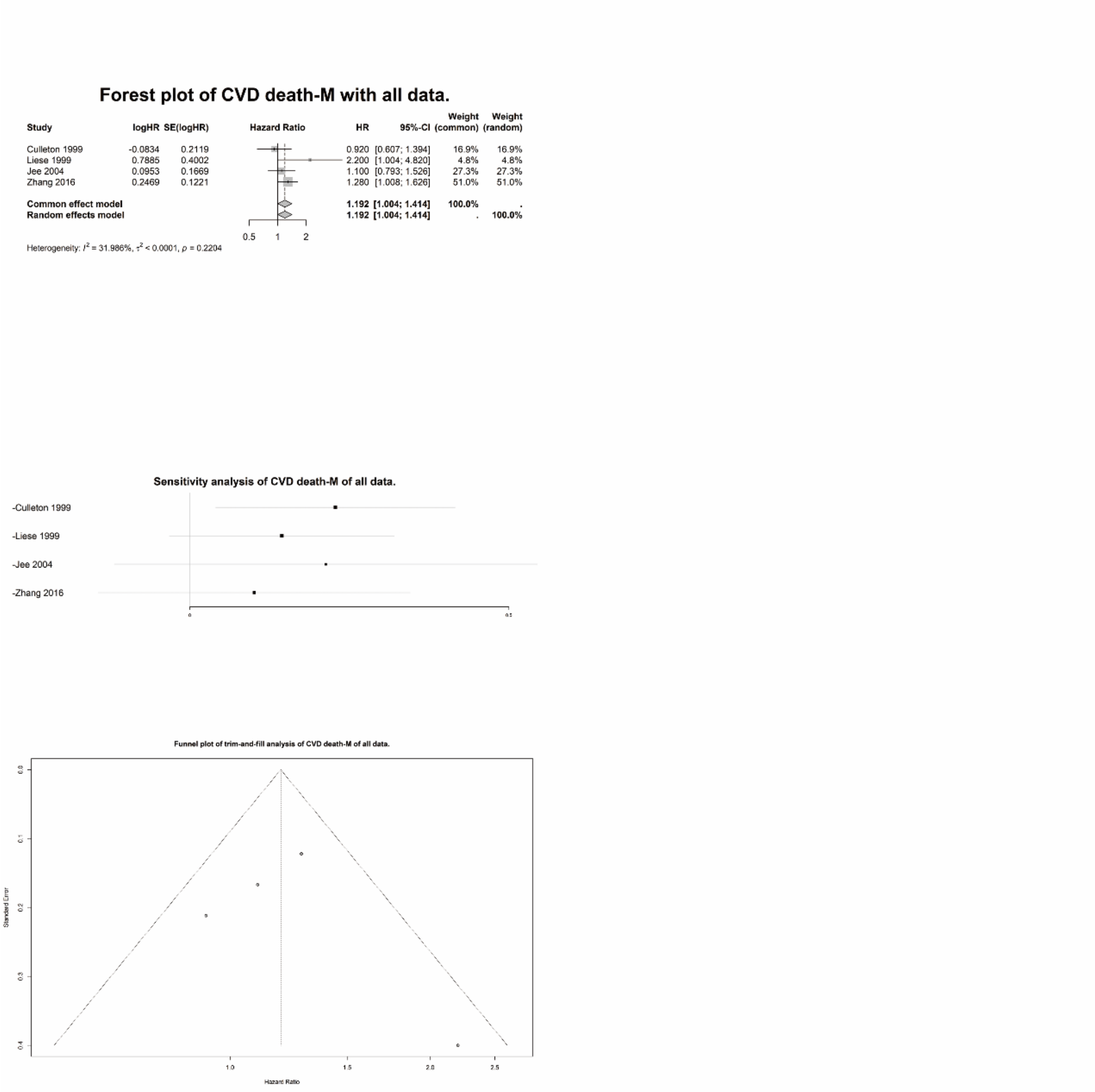


Figure S2-13. Forest plots, sensitivity analysis, and funnel plots of association between hyperuricemia and CVD death among male population, with the inclusion of quantile data. A total of 4 studies with 42,475 participants were included in the pooled estimation.

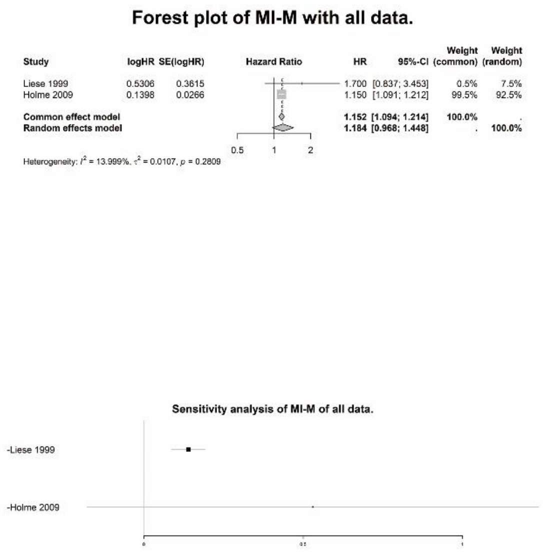


Figure S2-14. Forest plots, sensitivity analysis of association between hyperuricemia and MI among male population, with the inclusion of quantile data. A total of 2 studies with 222,252 participants were included in the pooled estimation.

## Forest plot of MI-F with all data.

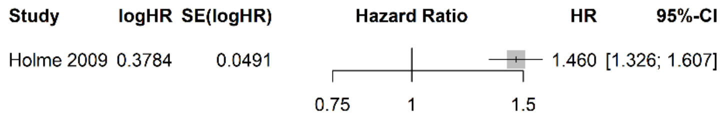


Figure S2-15. Forest plot of association between hyperuricemia and MI among female population. A total of 1 study with 196,556 participants were included in the pooled estimation.

**Supplementary Figures S3. Forest plots, sensitivity analysis, and funnel plots of association between increase of 1 unit or 1mg/dL of serum UA and cardiovascular outcomes.**

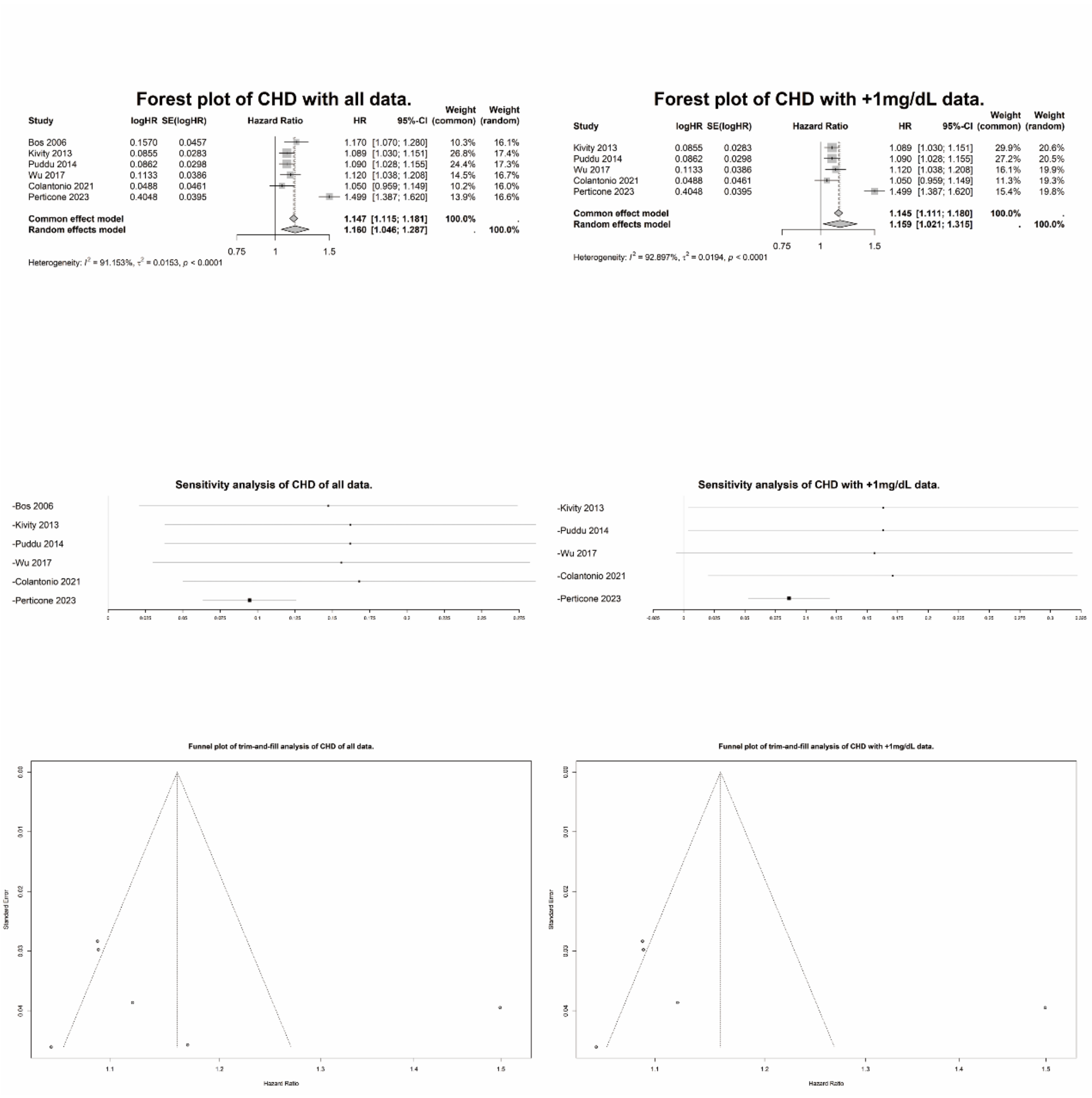


Figure S3-1. Forest plots, sensitivity analysis, and funnel plots of association between increase of 1 unit or 1mg/dL of serum UA and CHD. A total of 6 studies with 22,130 participants were included in the pooled estimation, while 5 studies with 17,745 participants were included for the studies with +1mg/dL uric acid data.

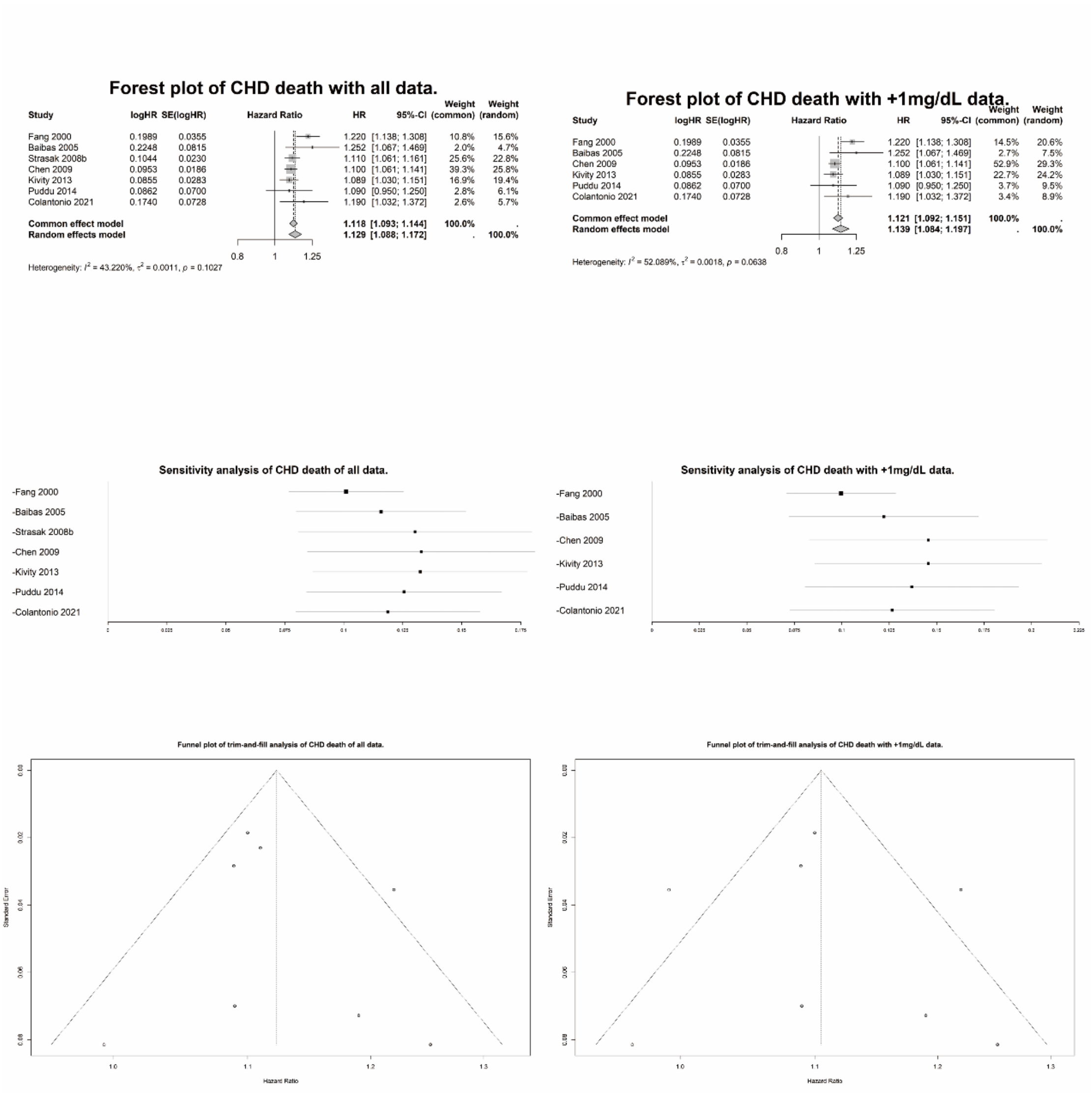


Figure S3-2. Forest plots, sensitivity analysis, and funnel plots of association between increase of 1 unit or 1mg/dL of serum UA and CHD death. A total of 7 studies with 140,035 participants were included in the pooled estimation, while 6 studies with 111,422 participants were included for the studies with +1mg/dL uric acid data.

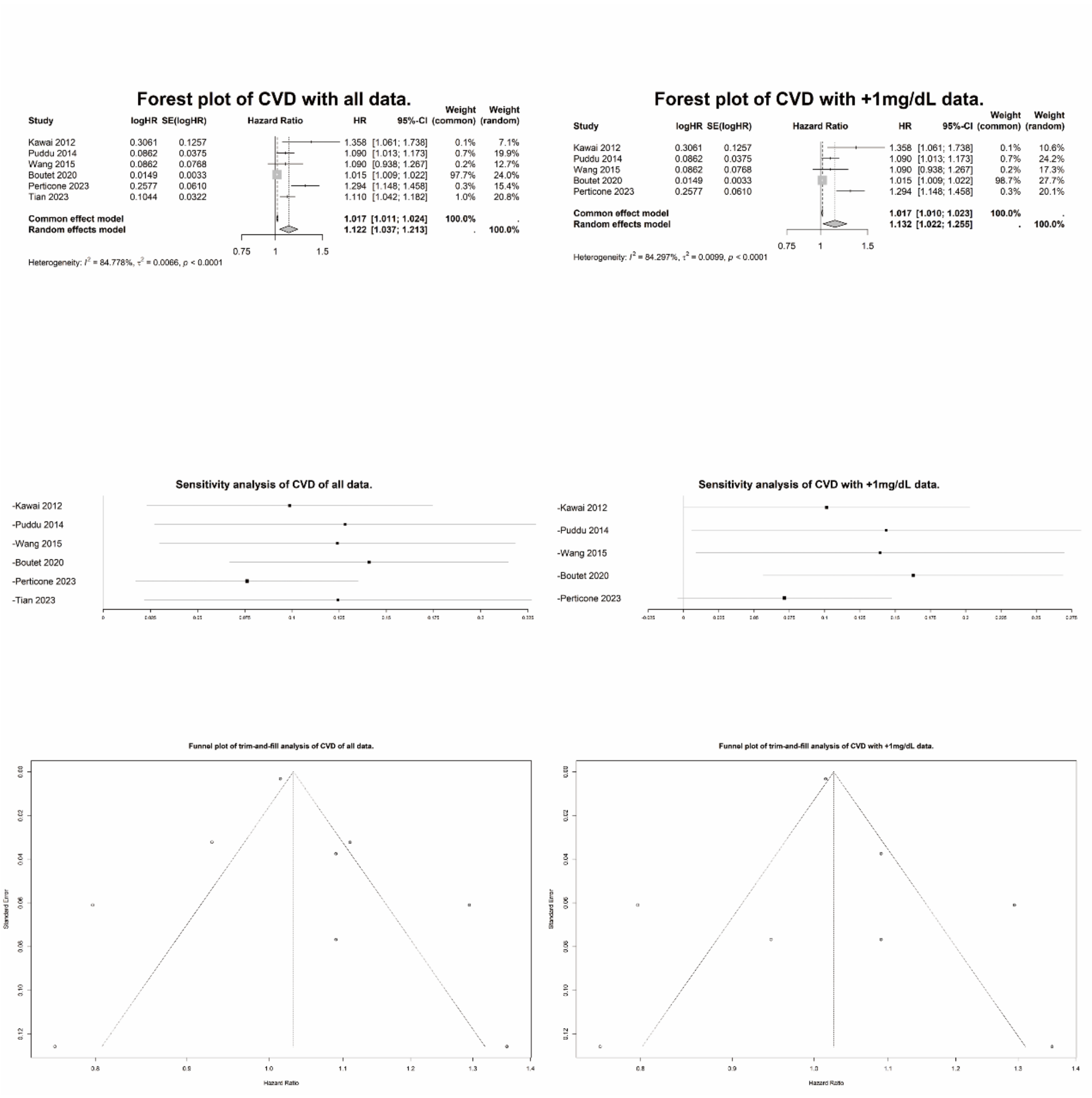


Figure S3-3. Forest plots, sensitivity analysis, and funnel plots of association between increase of 1 unit or 1mg/dL of serum UA and CVD. A total of 6 studies with 53,456 participants were included in the pooled estimation, while 5 studies with 28,172 participants were included for the studies with +1mg/dL uric acid data.

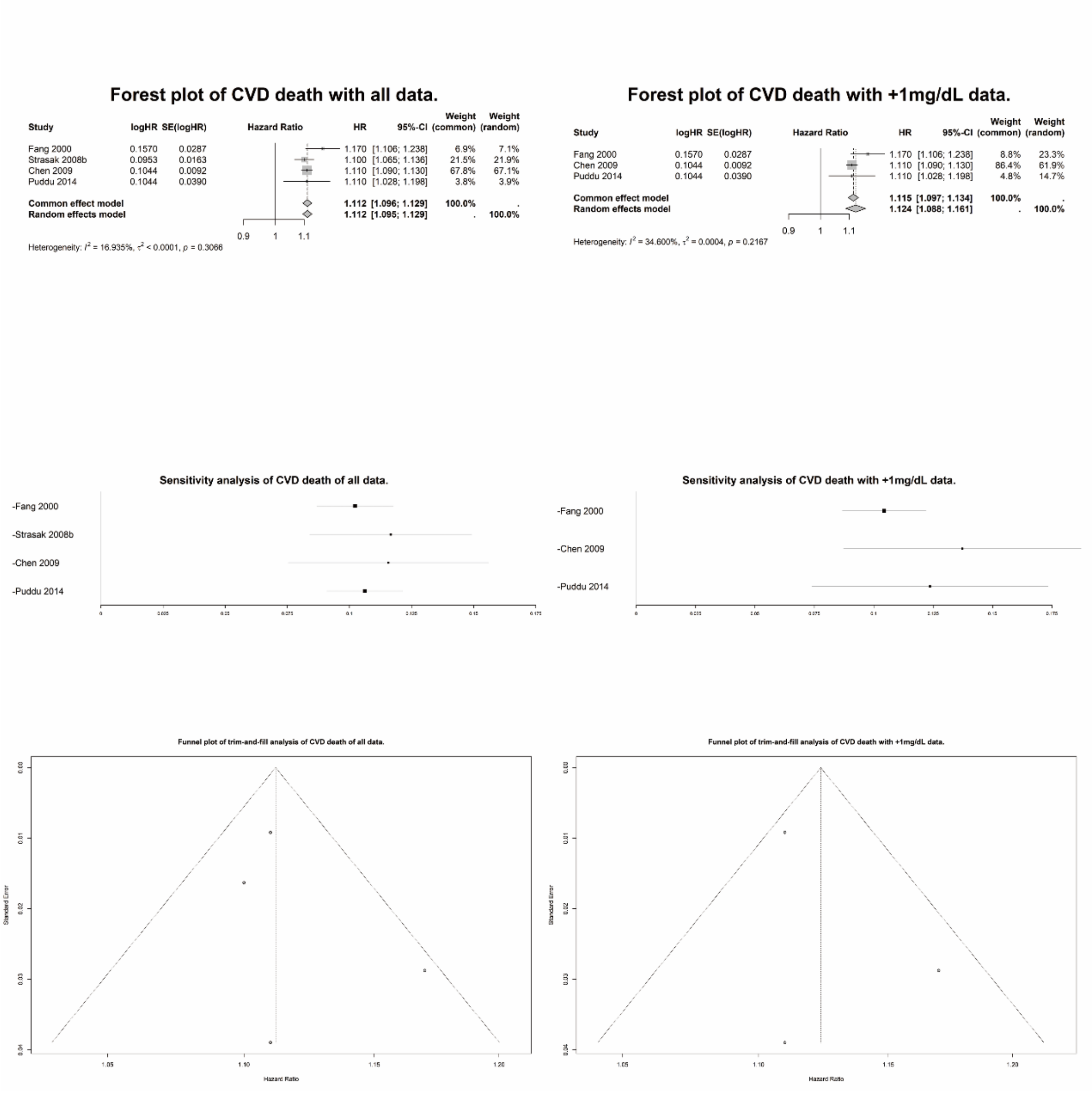


Figure S3-4. Forest plots, sensitivity analysis, and funnel plots of association between increase of 1 unit or 1mg/dL of serum UA and CVD death. A total of 4 studies with 127,820 participants were included in the pooled estimation, while 3 studies with 99,207 participants were included for the studies with +1mg/dL uric acid data.

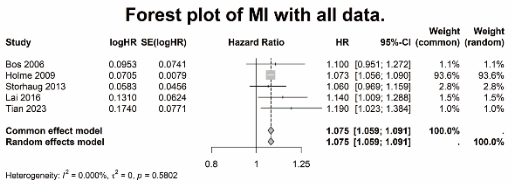


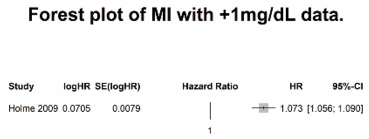


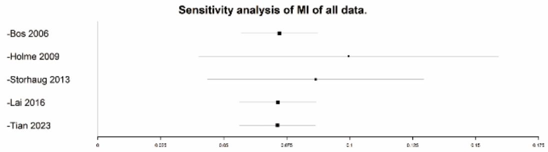


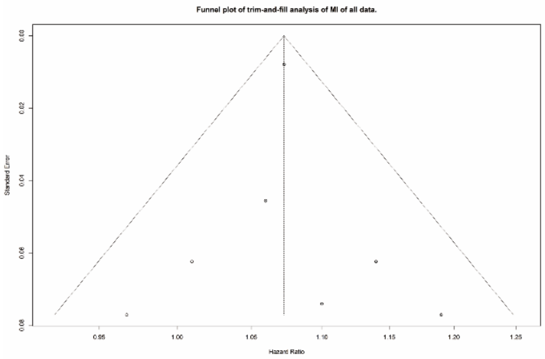


Figure S3-5. Forest plots, sensitivity analysis, and funnel plots of association between increase of 1 unit or 1mg/dL of serum UA and MI. A total of 5 studies with 469,166 participants were included in the pooled estimation, while 1 study with 417,734 participants were included for the studies with +1mg/dL uric acid data.

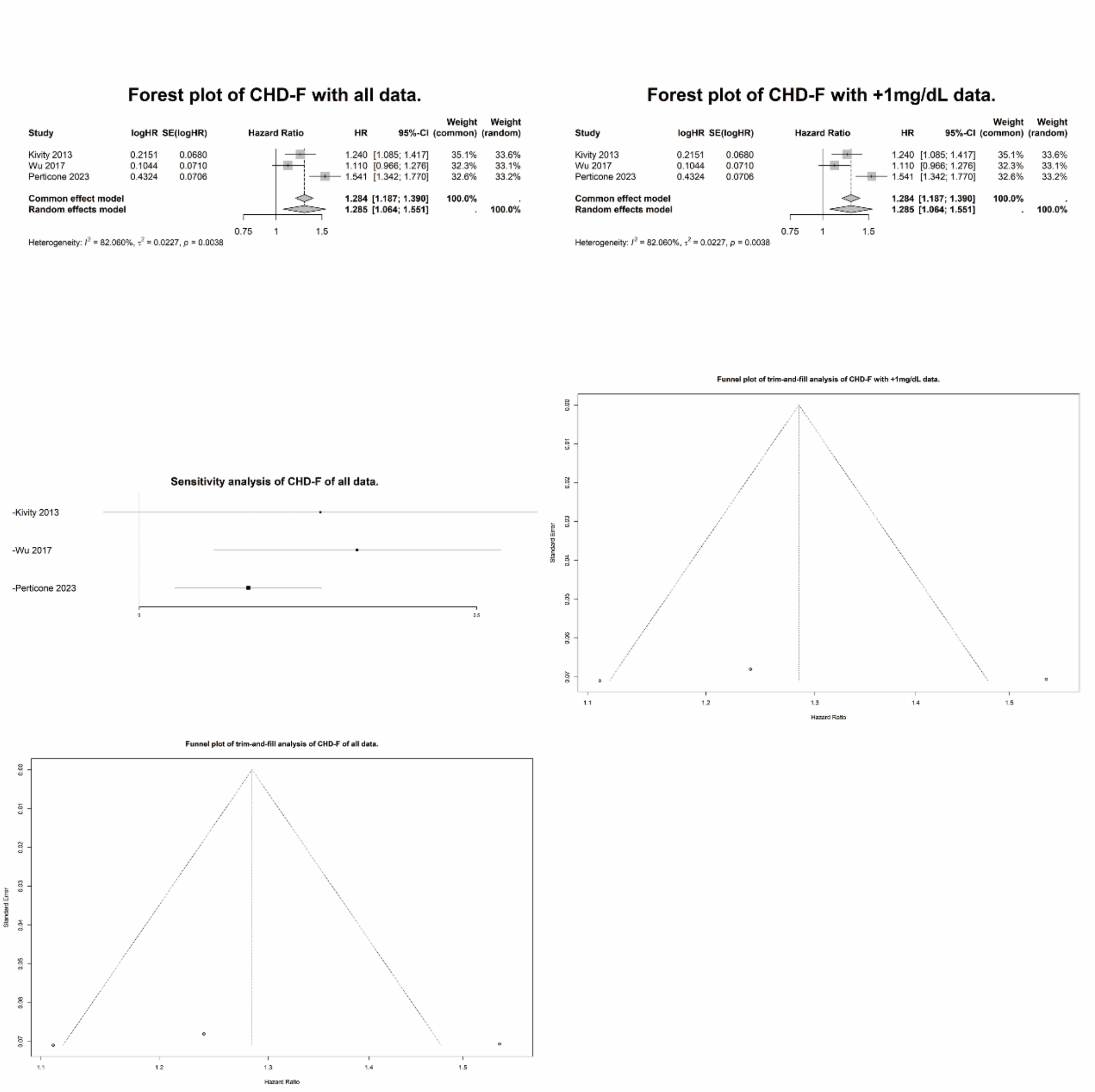


Figure S3-6. Forest plots, sensitivity analysis, and funnel plots of association between increase of 1 unit or 1mg/dL of serum UA and CHD among female population. A total of 3 studies with 4,327 participants were included in the pooled estimation, while 3 studies with 4,327 participants were included for the studies with +1mg/dL uric acid data.

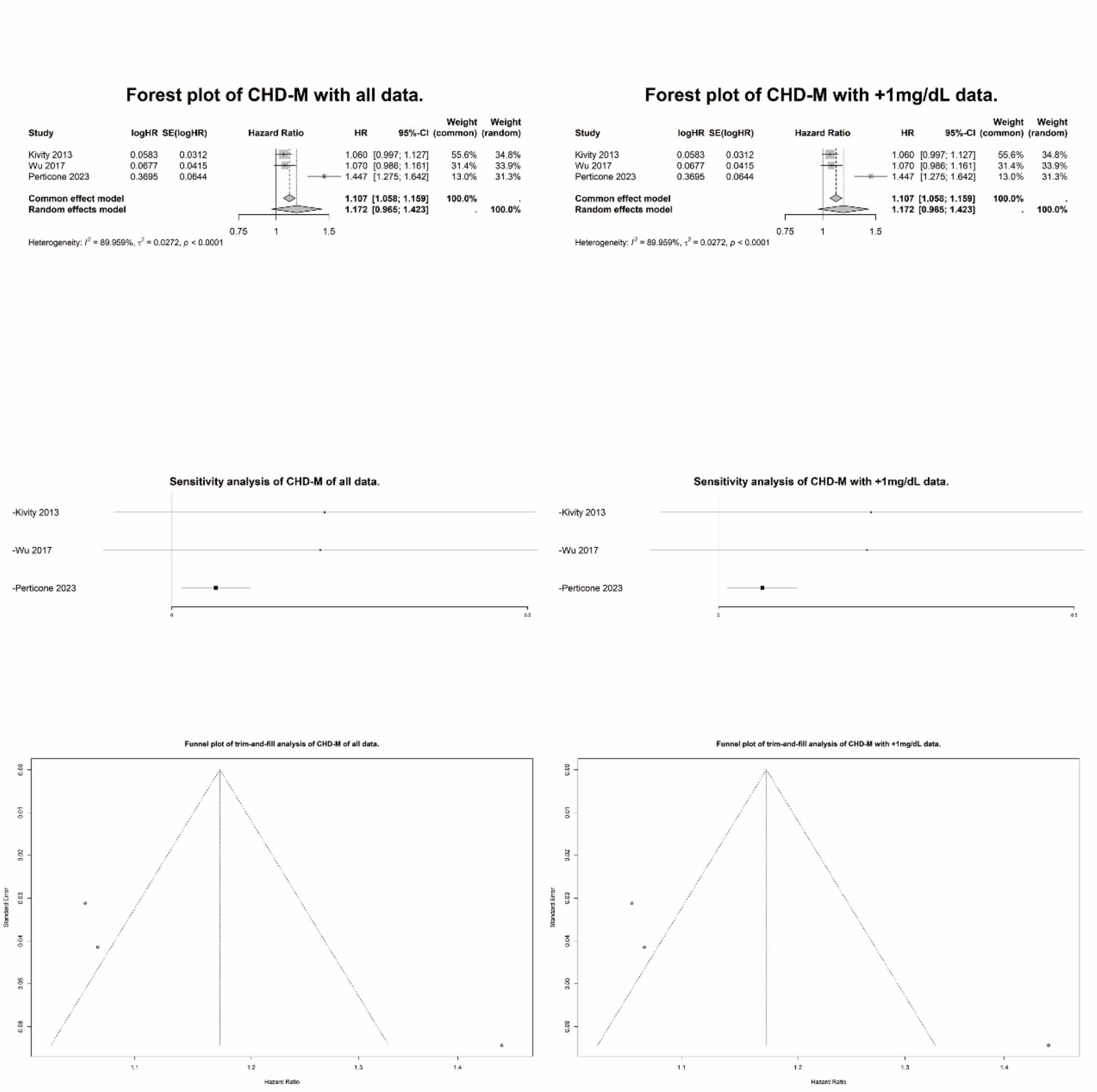


Figure S3-7. Forest plots, sensitivity analysis, and funnel plots of association between increase of 1 unit or 1mg/dL of serum UA and CHD among male population. A total of 3 studies with 8,604 participants were included in the pooled estimation, while 3 studies with 8,604 participants were included for the studies with +1mg/dL uric acid data.

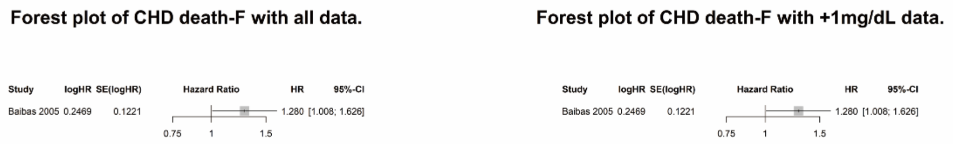


Figure S3-8. Forest plots of association between increase of 1 unit or 1mg/dL of serum UA and CHD death among female population. A total of 1 study with 646 participants were included in the pooled estimation, while 1 study with 646 participants were included for the studies with +1mg/dL uric acid data.

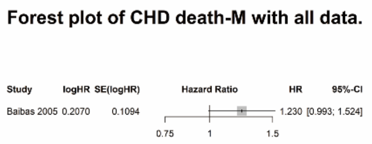


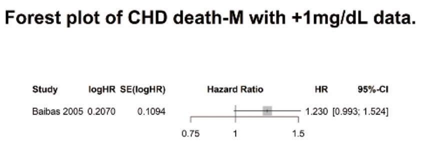


Figure S3-9. Forest plots, sensitivity analysis, and funnel plots of association between increase of 1 unit or 1mg/dL of serum UA and CHD death among male population. A total of 1 study with 504 participants were included in the pooled estimation, while 1 study with 504 participants were included for the studies with +1mg/dL uric acid data.

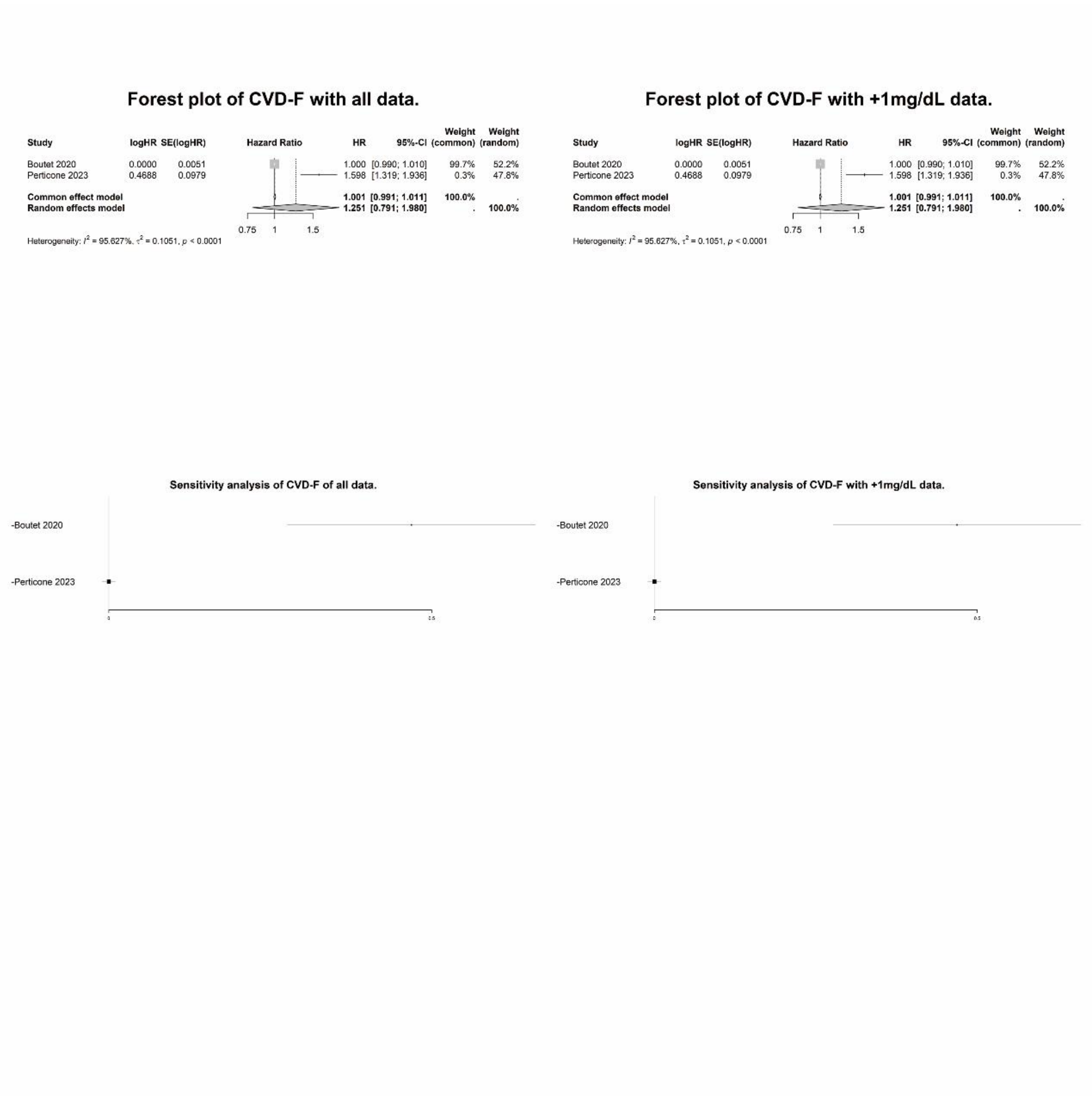


Figure S3-10. Forest plots, sensitivity analysis of association between increase of 1 unit or 1mg/dL of serum UA and CVD among female population. A total of 2 studies with 10,349 participants were included in the pooled estimation, while 2 studies with 10,349 participants were included for the studies with +1mg/dL uric acid data.

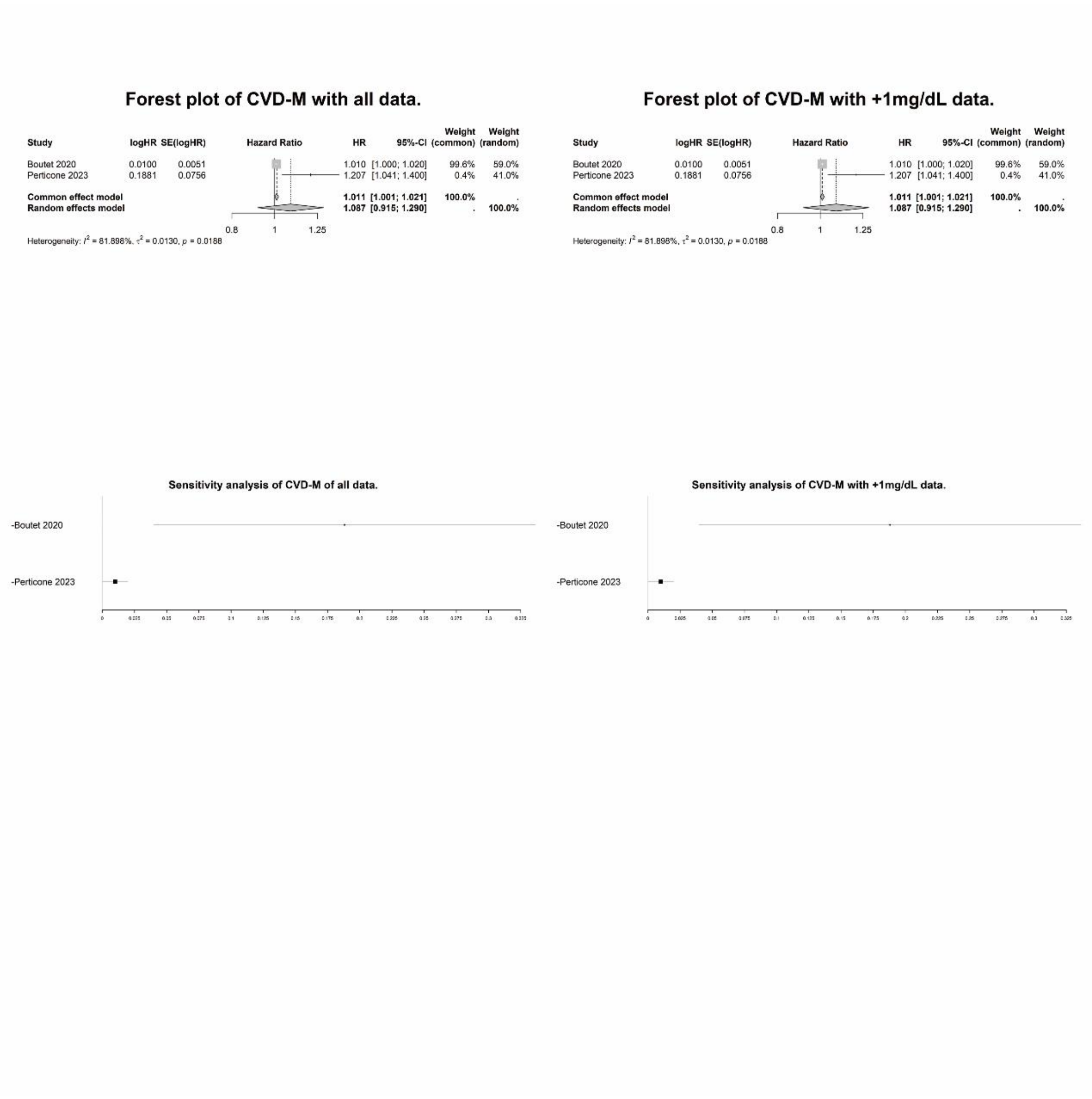


Figure S3-11. Forest plots, sensitivity analysis of association between increase of 1 unit or 1mg/dL of serum UA and CVD among male population. A total of 2 studies with 9,450 participants were included in the pooled estimation, while 2 studies with 9,450 participants were included for the studies with +1mg/dL uric acid data.

## Forest plot of CVD death-F with all data.

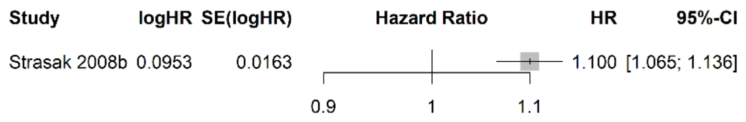


Figure S3-12. Forest plot of association between increase of 1 unit or 1mg/dL of serum UA and CVD death among female population. A total of 1 study with 28,613 participants were included in the pooled estimation.

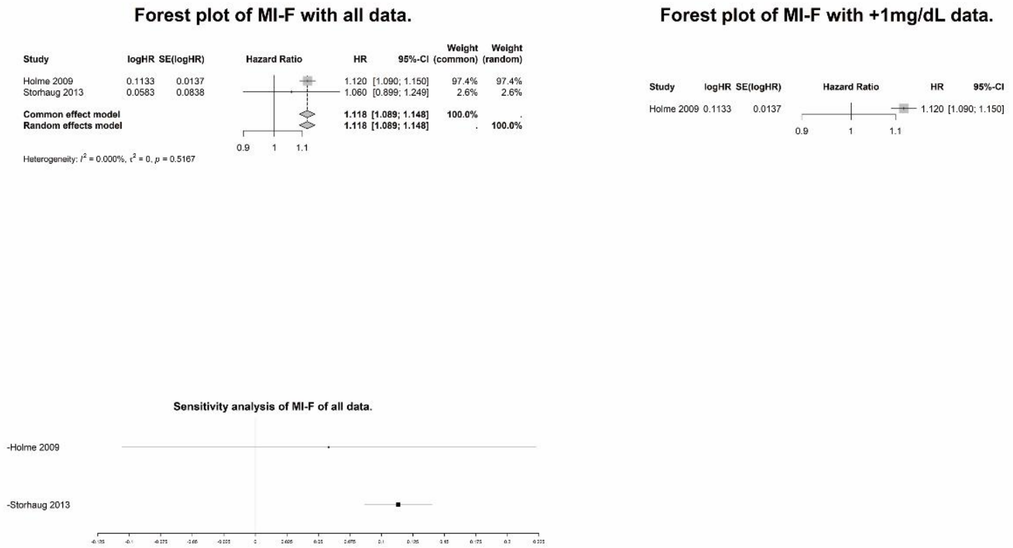


Figure S3-13. Forest plots, sensitivity analysis of association between increase of 1 unit or 1mg/dL of serum UA and MI among female population. A total of 2 studies with 199,560 participants were included in the pooled estimation, while 1 study with 196,556 participants were included for the studies with +1mg/dL uric acid data.

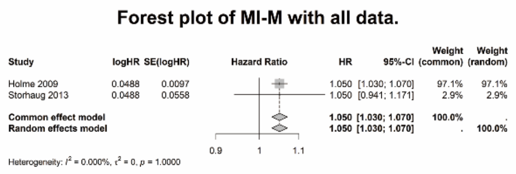


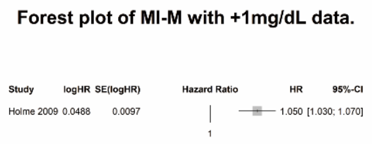


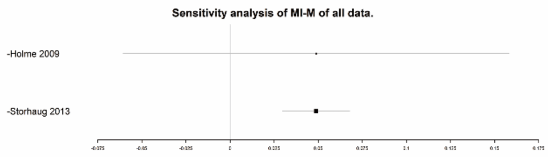


Figure S3-14. Forest plots, sensitivity analysis of association between increase of 1 unit or 1mg/dL of serum UA and MI among male population. A total of 2 studies with 223,874 participants were included in the pooled estimation, while 1 study with 221,178 participants were included for the studies with +1mg/dL uric acid data.

**Supplementary Figures S4. Restricted cubic spline regression plots of dose-response meta-analysis.**

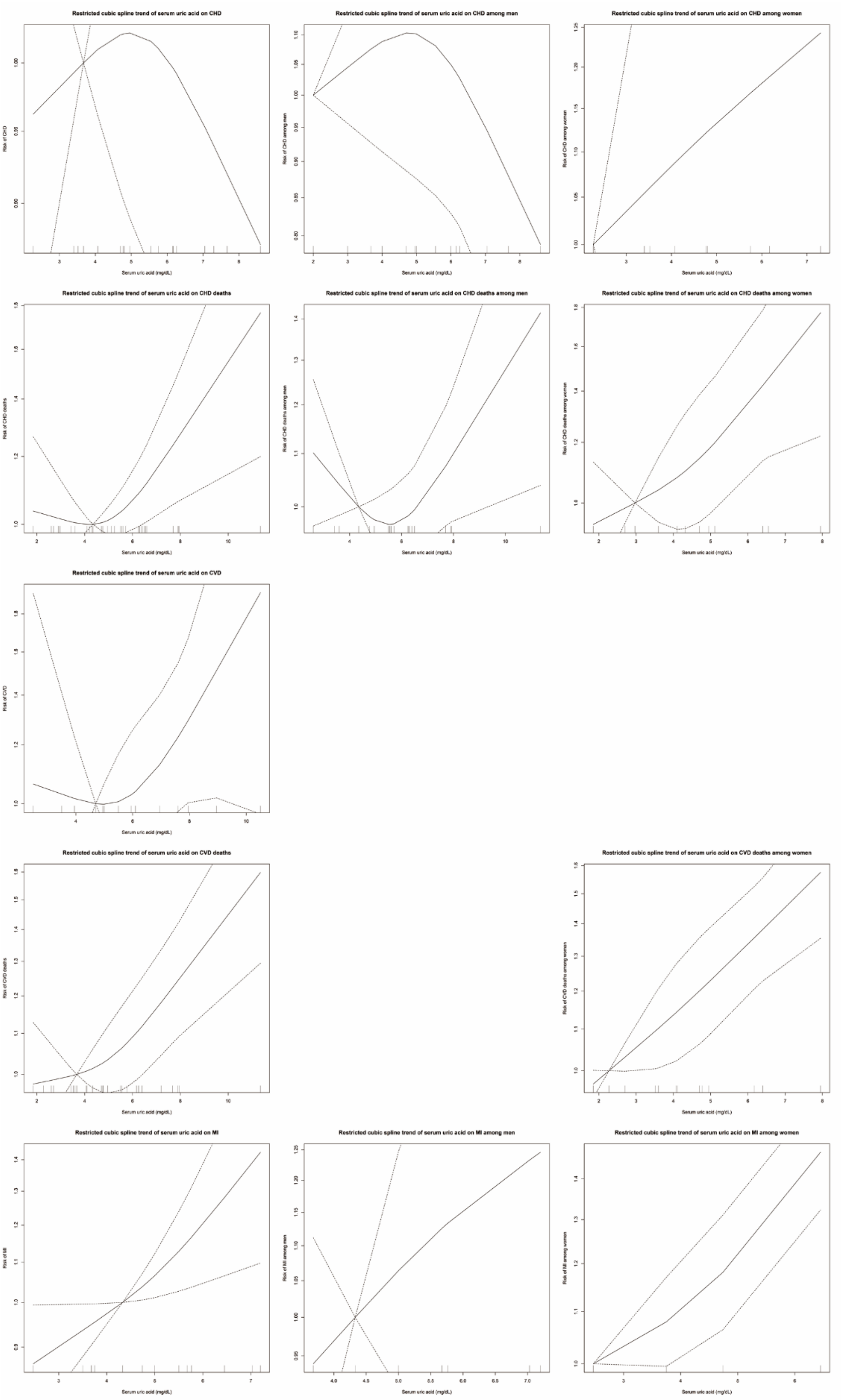

Supplement: S1 Appendix — (PDF) [file pone.0337091.s001.pdf]
