# Supplementary material for: Association of hyperuricemia with coronary heart disease and other cardiovascular outcomes: A systematic review and dose-response meta-analysis
Source: PLoS One. 2025 Nov 18;20(11):e0337091. doi: 10.1371/journal.pone.0337091 (PMC12626327; doi:10.1371/journal.pone.0337091)
Supplement: Attachment — Submitted filename: Response to reviewers.docx [file pone.0337091.s005.docx]

Dear editor,

We sincerely appreciate you and the reviewers for your patience and professional work on our manuscript (PONE-D-25-42229). We have read through comments carefully, made corrections, and responded to each comment in the below point-by-point. We believe that the quality of our revised manuscript has been improved with your suggestions. The point-by-point response is as below for your kindly review.

Thank you for your consideration. I look forward to hearing from you.

Sincerely,

Jingen Li,

Deputy Chief Physician,

Department of Cardiovascular Medicine, Dongzhimen Hospital, Beijing University of Chinese Medicine,

Haiyuncang 5, Dongcheng District,

Beijing, China, 100029.

lijingen198908@126.com

Yong Zhang,

Chief Pyhsician,

Dongzhimen Hospital, Beijing University of Chinese Medicine, Haiyuncang 5, Dongcheng District, Beijing, China, 100029;

The seventh affiliated hospital of Xinjiang Medical University, Qidaowan South Road 1986, Urumqi, Xinjiang, China, 830028.

zhangyong_tcm@163.com

October 11 2025

Additional Editor Comments :

In this manuscript, Lyu et al. present a systematic review and meta-analysis of observational studies evaluating the association between hyperuricemia and coronary heart disease (CHD). By systematically searching Medline, the Cochrane Library, Embase, and two trial registration databases from inception to June 30, 2025, the authors identified 42 articles corresponding to 39 unique studies, encompassing a total of 1,082,880 participants. The pooled results demonstrated a statistically significant association between hyperuricemia and increased risk of CHD, as well as its individual components (CHD death, cardiovascular death, myocardial infarction). The dose–response analyses further suggested a linear relationship between serum uric acid (UA) and cardiovascular disease (CVD) risk, and a U-shaped association between serum UA and risk of CVD death among men. Despite the relevance of the research question and the adherence to a registered protocol, the overall certainty of the evidence was limited by the observational study designs. Several key aspects require clarification and further development before the manuscript can be considered suitable for publication.

Response: We sincerely appreciate the editor and reviewers for their thorough and professional evaluation of our manuscript. In particular, we are grateful for the insightful guidance regarding heterogeneity, meta-regression, and the organization of the Supplementary Materials. These comments prompted us to reexamine all included studies and perform additional analyses. Although the overall conclusions remain largely unchanged, we are now more confident in their robustness and reliability. We believe that the constructive feedback and our corresponding revisions have substantially improved the quality of the manuscript. Please review.

Risk of Bias: Although the original protocol did not specify separate analyses for CHD-related outcomes, the authors expanded their work to include CHD death, CVD death, and myocardial infarction. While this approach is wisely comprehensive, it raises concerns regarding potential spin bias in relation to the primary outcome (CHD). Importantly, substantial heterogeneity was observed across included studies, arising from differences in association measures, uric acid assessment methods, and other study-level factors. The sources of this statistical heterogeneity should be explicitly examined and reported in relation to study characteristics and association measurements (HR,OR,RR). Furthermore, given the relevance of confounding factors in the relationship between hyperuricemia and CHD, meta-regression analyses should be performed at least for age, sex, and body mass index (BMI).

Response: Thanks for your comment. We have noticed this from the comments of reviewer 1 and reviewer 2. We have carefully reviewed all included studies, extracted necessary data, and conducted additional analyses. As we found, there was only 1 article involved the odds ratio (OR) to report their finding (Shiozaki 2013, published in Japanese), 6 involved the risk ratio (RR), 4 did not report, while the other 31 involved the hazard ratio (HR). The subgroup analysis of RR or HR was reported as Supplementary Material, and the heterogeneities of CHD death and CVD death from HR subgroup significantly decreased to 0%, while the other heterogeneities was still observed by assessing *I^2^*.

We also summarized data about the regions, the uric acid assessment methods, the count of adjusted factors, follow-up duration, BMI, age, and sex. We found 20 articles reported results from Asia, 13 from Europe, and 9 from North America. The descriptions on the uric acid assessment methods were not clear enough in most of the articles. With the help of Google, we found 11 articles used the uricase-peroxidase method, 6 used colorimetric method, 6 used phosphotungstic acid method, 5 used uricase method, and 14 with missing information. A total of 41 articles reported the adjusted factors in their statistical models, ranged from 0 to 24. For the follow-up duration, 26 articles reported the time range (from 4 to 27 years), 17 reported the average follow-up years (ranged from 2.8 to 16.8 years), 1 reported the median follow-up duration, and 1 with missing information. Considering the BMI data, 4 articles reported the BMI data of the event-group and nonevent-group, 3 reported categorized data but not mean with SD, 8 just reported the BMI data of the total population only, and 19 articles reported BMI data of the hyperuricemia population and reference population. A total of 35 articles reported the age of the included population, while only 1 article did not report the sex distribution. The above-mentioned information has been added to the tables of the manuscript or the Supplementary Materials. We found only 14 articles with available data of age, sex, and BMI. Therefore, we found it hard to perform satisfactory meta-regression.

We performed meta-regressions with age, sex, and BMI, for CHD, CHD death, CVD, CVD death, and MI, for the pooled estimations comparing participants with hyperuricemia with controls. Among the 5 outcomes, only the data of CHD death and MI were available, and found that higher proportion of male might increase the risk of CHD death among participants with hyperuricemia (β = -0.91, 95%CI -1.66 to -0.15, p = 0.018). No significant association was found for MI. When performing meta-regression without BMI, we found tiny increasing risk of CVD among male participants with hyperuricemia (β = 0.00, 95%CI -0.01 to 0.00, p = 0.043). We also performed meta-regression to test the potential confounding factors on the association between per unit increase of uric acid and the risk of cardiovascular outcome. For all 5 outcomes, BMI data was not enough to perform meta-regression, while no significant association was found on age or sex.

In conclusion, we have tried our best to explore the possible source of heterogeneity. However, through additional subgroup analyses and meta-regressions, we obtained only limited findings, which did not sufficiently explain the majority of the observed heterogeneity. We have added the above findings to the manuscript or the Supplementary Material, and added text in the Discussion section to clearly describe and report our findings to the readers. I hope this would be helpful for the further investigators. Please review.

Discussion: The Discussion would benefit from additional depth. Specifically, the clinical relevance of the findings should be contextualized in light of potential therapeutic strategies. Moreover, possible mechanisms underlying the observed U-shaped association between serum uric acid and CVD mortality in men should be elaborated.

Response: Thanks for your comment. We have searched the PubMed for articles on potential therapeutic strategies. We found that most of the articles in this field involved participants with cardiovascular disease and hyperuricemia or gout (has been discussed in our Discussion section), but not population with hyperuricemia and free of cardiovascular disease. Thus, we added the following text: “*Currently, there is a substantial body of research on urate-lowering therapy for gout[91], but studies specifically addressing the management of asymptomatic hyperuricemia remain limited. Our study reports that hyperuricemia is associated with an increased risk of cardiovascular outcomes, yet it remains unclear whether urate-lowering interventions in this high-risk population would confer cardiovascular benefits, and which urate-lowering therapies would be better. We encourage researchers to conduct large-scale, long-term cohort studies in this field to evaluate the urate-lowering effects of various pharmacological and non-pharmacological interventions and to assess their long-term impact on cardiovascular outcomes in high-risk populations, thereby providing evidence for future clinical practice.*”

We carefully reviewed the literature and attempted to interpret the finding of U-shaped association. However, as presented in our newly added Supplementary Figure S4, a similar U-shaped association was also observed for CHD mortality in men (p>0.05), whereas the risk of CHD itself exhibited an inverted U-shaped association (p>0.05). These patterns were not observed among women. Although we explored potential explanations for the U-shaped association, we were unable to further clarify the reasons for the inverted U-shaped relationship for CHD risk in men, or why a similar pattern was not observed in women. We acknowledge this limitation in the Discussion section and emphasize that our findings are based on limited data, highlighting the need for further studies to validate these results. For this reason, this finding was not emphasized in the Conclusion section or the Abstract section. We also anticipate that future mechanistic investigations will provide a better understanding of the underlying causes of the U-shaped association. We have added the following text in the Discussion section: “*Our study identified a U-shaped association between serum UA levels and the risk of CVD mortality among men. As shown in Supplementary Figure S4, a similar U-shaped pattern was also observed for CHD mortality in men (p>0.05), whereas the risk of CHD itself demonstrated an inverted U-shaped association (p>0.05). Interestingly, these associations were not observed among women. Similar pattern has been observed from other studies as well, including kidney failure and mortality in chronic kidney disease[73], composite outcome of COVID-19 patients[74], etc. The relationship between hyperuricemia and endothelial dysfunction or vascular injury has been well recognized[75]. Some studies have suggested that uric acid possesses physiological antioxidant[76] and immunomodulatory properties[77], and that excessively low UA levels may impair free radical scavenging capacity and reduce immune regulation, ultimately leading to endothelial damage. This mechanism might explain the increased CVD mortality observed in men with low uric acid levels. However, it does not account for the inverted U-shaped association with CHD risk in men, nor the absence of similar patterns in women. It should be acknowledged that the number of original studies included in the dose-response meta-analysis was limited, which may affect the stability of the results. Further studies with high-quality are warranted to verify or correct our findings. We also anticipate that future research on uric acid will provide a more comprehensive understanding of its dual effects at different concentration levels.*” Please review.

Language and Style: Minor typographical and grammatical errors should be carefully revised throughout the manuscript. For example, in the Abstract, the sentence “Among them, 2 article was assess as ‘Very high risk of bias’, 8 ‘High risk of bias’, 2 ‘Some concerns’” requires correction for grammar and style. A comprehensive language edit is recommended.Please also consider that Numbers with four or more digits should have a comma inserted at the thousands place for readability, such as 1,234 or 10,000.

Response: Thanks for the comment on language and style. We have carefully revised the whole text accordingly. The commas have been inserted. We have also invited a researcher (Yan Li from the Yong Loo Lin School of Medicine, National University of Singapore) to help us revise the manuscript. We hope the quality of language has been improved with her help. Please review.

Reporting Standards and Methodological Transparency: In addition to PRISMA reporting, adherence to the MOOSE guidelines (Stroup et al., JAMA 2000) should be explicitly documented, in line with the reporting standards referenced in the registered protocol. This will strengthen the methodological transparency and ensure consistency with accepted standards for meta-analyses of observational studies.

Response: Thanks for the comment. We have actually conducted this systematic review according to the MOOSE guidelines, but forgot to mention it in the manuscript. We are really sorry for this mistake. We have revised the text in the first paragraph of the Methods section as “*……and the Meta-analysis Of Observational Studies in Epidemiology (MOOSE) guidelines [27] ……*”, and uploaded the MOOSE checklist PDF file (MOOSE checklist.pdf) as attachment file. Please review.

Review Comments to the Author

Please use the space provided to explain your answers to the questions above. (Please upload your review as an attachment if it exceeds 20,000 characters)

Reviewer #1: The present is an intetesting paper aiming to evalaute impact of uricemia on CV outcomes. It is sound from a methodological point of view.

Response: Thanks for your valuable comments on our manuscript. We appreciate your patience on our work. We have carefully revised our manuscript and performed additional analyses following your guidance. We believe that the quality of the manuscript has been improved after this revision. Please review.

Some issues should be addressed

1) metaregression analysis for age, gender and BMI should be performed

Response: Thanks for your comment. Following the comments from you and the reviewer 2, we extracted more information from the articles to perform subgroup analyses and meta-regressions.

We have summarized data about the regions, the uric acid assessment methods, the count of adjusted factors, follow-up duration, BMI, age, and sex. We found 20 articles reported results from Asia, 13 from Europe, and 9 from North America. The descriptions on the uric acid assessment methods were not clear enough in most of the articles. With the help of Google, we found 11 articles used the uricase-peroxidase method, 6 used colorimetric method, 6 used phosphotungstic acid method, 5 used uricase method, and 14 with missing information. A total of 41 articles reported the adjusted factors in their statistical models, ranged from 0 to 24. For the follow-up duration, 26 articles reported the time range (from 4 to 27 years), 17 reported the average follow-up years (ranged from 2.8 to 16.8 years), 1 reported the median follow-up duration, and 1 with missing information. Considering the BMI data, 4 articles reported the BMI data of the event-group and nonevent-group, 3 reported categorized data but not mean with SD, 8 just reported the BMI data of the total population only, and 19 articles reported BMI data of the hyperuricemia population and reference population. A total of 35 articles reported the age of the included population, while only 1 article did not report the sex distribution. The above-mentioned information has been added to the tables of the manuscript or the Supplementary Materials. We found only 14 articles with available data of age, sex, and BMI. Therefore, we found it hard to perform satisfactory meta-regression, especially for BMI.

We performed meta-regressions with age, sex, and BMI, for CHD, CHD death, CVD, CVD death, and MI, for the pooled estimations comparing participants with hyperuricemia with controls. Among the 5 outcomes, only the data of CHD death and MI were available, and found that higher proportion of male might increase the risk of CHD death among participants with hyperuricemia (β = -0.91, 95%CI -1.66 to -0.15, p = 0.018). No significant association was found for MI. When performing meta-regression without BMI, we found tiny increasing risk of CVD among male participants with hyperuricemia (β = 0.00, 95%CI -0.01 to 0.00, p = 0.043). We also performed meta-regression to test the potential confounding factors on the association between per unit increase of uric acid and the risk of cardiovascular outcome. For all 5 outcomes, BMI data was not enough to perform meta-regression, while no significant association was found on age or sex.

2) more data about baseline features of each study should be added

Response: Thanks for your comment. We have established the data extraction plan referring to the previous systematic reviews in this field. Your suggestions have made us realize that the information we have summarized is insufficient. Following your suggestion, we have added as much information as possible, including the BMI data, outcome type (HR, OR, or RR), uric acid assessment methods, original study start year, definition of outcome, and judgment method for outcome, etc., into Table 1 or Table S1. As we described in the response of your comment 1), most of the studies reported incomplete information, and we have tried our best to comprehensively summarize them. Please review.

3) recently bempedoic acid has emerged as a relevant therapy to reduce cholesterol levels, although increasing that of uric acid. Authors should comment on this (quote and comment on PMID: 38017541)

Response: Thanks for your comment. We have carefully read this article and discussed among all of us. To the best of our knowledge, this article demonstrated the efficacy and safety of bempedoic acid on MACE, except for an increased risk of gout. We have cited this article to support our study (The current Reference 18 of the revised manuscript). We have also added this article to our collection, as we believe it will also provide valuable support for our future research findings. This study once again highlights the importance of monitoring uric acid levels and gout symptoms when using lipid-lowering therapies in clinical practice.

Reviewer #2: This manuscript addresses an important clinical and epidemiological question regarding the association between hyperuricemia and cardiovascular outcomes. The authors conducted a systematic review and dose-response meta-analysis with registration in PROSPERO and application of ROBINS-E and GRADE, which is commendable. The study is generally well-structured and the analyses are extensive, including sex-specific and dose-response models.

Response: We sincerely appreciate your thorough, comprehensive, and professional review of our manuscript. Following your comments, we carefully reread all included studies, re-extracted the necessary data, conducted new analyses, and compared the new results with our previous findings. We then made substantial revisions to the manuscript accordingly. We believe that the revisions according to your suggestions have greatly improved the quality of our work. We kindly invite you to review the revised manuscript.

However, I suggest to adress the following points before this work can be considered for publication.

1. While the outcomes are indeed defined in both the main manuscript and the supplementary material, there is considerable heterogeneity in how individual studies defined them. Some cohorts relied on ICD codes, others on clinical/ECG confirmation, and some even included procedures such as CABG or PCI as part of the endpoint. Older cohorts often applied broader clinical definitions such as “typical angina” or “vascular disease.” This variability limits the comparability of studies and likely contributes to the high heterogeneity observed in pooled analyses. The authors should more explicitly acknowledge this as a major limitation. Furthermore, according to the registered protocol, the primary outcome was CHD, whereas the manuscript expands the scope to include additional outcomes (CVD, CVD death, MI). This expansion should be transparently reported as a deviation from the original protocol.

Response: Thanks for your detailed suggestion. The definition of the outcomes might cause considerable heterogeneity. To solve this problem, we have reviewed all of the included articles again, and extracted the exact definition of the outcomes, as well as the way of the researchers to determine the outcomes, and presented as the revised Supplementary Table S1. We found that it is impossible to solve this heterogeneity in the current systematic. Thus, we have followed your guidance and revised the text in the limitation paragraph (The last paragraph of the Discussion section) as following: “*…… substantial and potentially irreducible heterogeneity may exist among the included studies due to variations in the definitions of identical outcome measures, differences in diagnostic criteria for the same disease, updates in diagnostic codes, and discrepancies in how outcome events were ascertained (see Supplementary Table S1). These factors are consistent with the heterogeneity observed in our data. For the same reason, ……*”. Please review.

We have added text to transparently reported our deviation from the original protocol, including the additional outcomes (“*……, although these secondary outcomes were not mentioned in our study protocol, and should be regarded as the deviation to our protocol ……*”), and the meta-regression to detect the potential confounding factors instead of performing subgroup analysis: *We conducted meta-regression to identify potential factors that might be associated with the risk of cardiovascular outcomes. This was not prespecified in our study protocol[25], representing a deviation from the original plan. Given the incomplete reporting of original data in the included studies (Table 1 and Table S1) and the relatively small number of studies contributing to each meta-analysis, only three variables, age, sex, and body-mass index (BMI), were included in the meta-regression. For the analysis based on the increasing unit of serum UA, data availability allowed inclusion of only age and sex; therefore, we performed additional meta-regression using these two variables in the comparison between hyperuricemia and control groups as a sensitivity analysis. Considering the high proportion of missing data, no imputation was performed; instead, unavailable data were directly excluded from the analysis.* Please review.

2. Some analyses, particularly the dose–response models, report very high heterogeneity (I² >90%). While subgroup analyses by sex were conducted, this is insufficient to explain such variability. Further exploration using meta-regression (e.g., by age distribution, mean follow-up duration, study region, publication decade, and level of confounder adjustment) would strengthen the analysis and provide more insight into sources of heterogeneity.

Response: Thanks for your comment. We have noticed this from the comments of you and reviewer 1. We have carefully reviewed all included studies, extracted necessary data, and conducted additional analyses. As we found, there was only 1 article involved the odds ratio (OR) to report their finding (Shiozaki 2013, published in Japanese), 6 involved the risk ratio (RR), 4 did not report, while the other 31 involved the hazard ratio (HR). The subgroup analysis of RR or HR was reported as Supplementary Material, and the heterogeneities of CHD death and CVD death from HR subgroup significantly decreased to 0%, while the other heterogeneities was still observed by assessing *I^2^*.

We also summarized data about the regions, the uric acid assessment methods, the count of adjusted factors, follow-up duration, BMI, age, and sex. We found 20 articles reported results from Asia, 13 from Europe, and 9 from North America. The descriptions on the uric acid assessment methods were not clear enough in most of the articles. With the help of Google, we found 11 articles used the uricase-peroxidase method, 6 used colorimetric method, 6 used phosphotungstic acid method, 5 used uricase method, and 14 with missing information. A total of 41 articles reported the adjusted factors in their statistical models, ranged from 0 to 24. For the follow-up duration, 26 articles reported the time range (from 4 to 27 years), 17 reported the average follow-up years (ranged from 2.8 to 16.8 years), 1 reported the median follow-up duration, and 1 with missing information. Considering the BMI data, 4 articles reported the BMI data of the event-group and nonevent-group, 3 reported categorized data but not mean with SD, 8 just reported the BMI data of the total population only, and 19 articles reported BMI data of the hyperuricemia population and reference population. A total of 35 articles reported the age of the included population, while only 1 article did not report the sex distribution. The above-mentioned information has been added to the tables of the manuscript or the Supplementary Materials. We found only 14 articles with available data of age, sex, and BMI. Therefore, we found it hard to perform satisfactory meta-regression.

We performed meta-regressions with age, sex, and BMI, for CHD, CHD death, CVD, CVD death, and MI, for the pooled estimations comparing participants with hyperuricemia with controls. Among the 5 outcomes, only the data of CHD death and MI were available, and found that higher proportion of male might increase the risk of CHD death among participants with hyperuricemia (β = -0.91, 95%CI -1.66 to -0.15, p = 0.018). No significant association was found for MI. When performing meta-regression without BMI, we found tiny increasing risk of CVD among male participants with hyperuricemia (β = 0.00, 95%CI -0.01 to 0.00, p = 0.043). We also performed meta-regression to test the potential confounding factors on the association between per unit increase of uric acid and the risk of cardiovascular outcome. For all 5 outcomes, BMI data was not enough to perform meta-regression, while no significant association was found on age or sex.

For the dose-response models, we carefully reviewed the manual of the *dosresmeta* package, and failed to find the method to conduct meta-regression on variables other than dose. We further extracted data from CHD-related studies but found the datasets incomplete. Even when including only age and sex as covariates, the available data were insufficient to perform meta-regression (7 articles included, but only 4 articles with complete data, while meta-regression requires at least 5 articles for 4 variables: dose, dose^2^, age, sex). Therefore, we were unable to supplement this part of the complementary analysis. To solve this issue, we have added text in the Discussion section.

In summary, we made every effort to explore the potential sources of heterogeneity. However, through additional subgroup analyses and meta-regressions, we obtained only limited findings, which did not sufficiently explain the majority of the observed heterogeneity. We have added the above findings to the manuscript or the Supplementary Material, and added text in the Discussion section to clearly describe and report our findings to the readers. I hope this would be helpful for the further investigators. Please review.

3. The GRADE assessments indicate that the overall certainty of evidence is low to very low. It would be advisable to include a stronger disclaimer regarding the potential for residual confounding and the inherent limitations of observational study designs.

Response: Thanks for the comment. To the best of our knowledge, we preferred to report a more rigorous and conservative result by a systematic review and meta-analysis; therefore, we applied strict criteria when assessing the quality of the evidence. According to your comment, we have added text addressing the potentially residual confounding, the inherent limitations of observational study design, and the relatively low certainty of evidence in the Discussion section as below: “*Using the GRADE tool, the quality of evidence was rated as low for CHD and very low for the other cardiovascular outcomes. It is important to emphasize that, due to the inherent limitations of observational studies, such as susceptibility to confounding factors and biases, they cannot control for bias through randomization as randomized controlled trials (RCTs) do. Therefore, the initial level of evidence for observational studies starts at low rather than high[86]. In our systematic review, several original studies were rated as having a high risk of bias, and substantial unexplained heterogeneity was also observed as above-mentioned. As described earlier, subgroup analyses and meta-regressions were conducted but could not adequately account for the heterogeneity. Although we performed a dose–response meta-analysis and other complementary analyses, residual confounding between original studies could not be fully eliminated, and the quality of evidence could not be upgraded. To our knowledge, this systematic review is the first to apply the GRADE framework to evaluate the quality of evidence regarding the association between hyperuricemia and CHD and other cardiovascular outcomes. Our findings highlight the need for cautious interpretation of the observed association between hyperuricemia and the clinical outcomes. Future original studies with higher methodological quality are warranted to provide more robust evidence to support clinical practice.*” Please review.

4. The Supplementary Table S1 shows wide variability in cut-offs (5.2–7.7 mg/dL, sex-specific thresholds, SD-based definitions). This heterogeneity undermines comparability and external validity. The included studies also used diverse uric acid measurement methods (enzymatic, phosphotungstic, colorimetric, or unreported). This introduces potential misclassification bias. The impact of assay heterogeneity could be discussed.

Response: Thanks for the comment. As mentioned above, we have extracted adding data from included articles, and revised the manuscript and Tables accordingly. Considering the uric adic measurement methods, we found 11 articles used the uricase-peroxidase method, 6 used colorimetric method, 6 used phosphotungstic acid method, 5 used uricase method, and 14 with missing information. Due to the lack of enough original studies, we failed to perform meta-regression by different measurement methods or different cut-offs. To address this limitation, we have emphasized it in the Discussion section. Heterogeneity may arise from multiple potential sources, including the type of statistical measurements, the study initiation or publication decades, regions, uric acid assessment methods, cut-offs of hyperuricemia, definition of each cardiovascular outcomes, and the approach to determine outcome events, etc. The original fourth paragraph of the Discussion section briefly addressed possible sources of heterogeneity; we have now expanded this section to incorporate a detailed discussion of the above factors. The text is as below: “…… *In addition to sex, we also conducted subgroup analyses by separating studies that reported results using HRs and those using RRs. We found that heterogeneity was substantially reduced in several outcomes reported with HRs, whereas no such reduction was observed in RR-reported results. This partially reveals one of the potential sources of heterogeneity. Notably, as shown in Supplementary Table S1, all studies published in the past decade have reported their results using HRs, which may help to partially control inter-study heterogeneity in the further systematic reviews. Furthermore, we observed considerable variation in how different studies defined the same cardiovascular outcomes. For example, CVD could refer to any combination of fatal or nonfatal CHD, MI, stroke, or other vascular conditions in different original studies. There were also discrepancies in disease coding systems, ranging from ICD-7 to ICD-10. Additionally, the methods used to determine whether an outcome event occurred varied across studies, including assessments based on medical records, self-reports by participants or family members, or clinical symptoms and auxiliary examinations. Decisions were made by different numbers of investigators or specialized committees in different original studies. Besides, the measurement of serum uric acid also differed among studies, employing diverse methods such as the uricase–peroxidase method, colorimetric method, phosphotungstic acid method, uricase method, or unreported techniques. The differences in measurement methods and cut-off values for defining hyperuricemia inevitably contributed to between-study heterogeneity. Finally, some sources of heterogeneity may be difficult to avoid, such as variations in study region, human race, sex distribution, age, and BMI, all of which can introduce residual and unresolvable heterogeneity. We recommend that future original studies take these factors fully into consideration and strive to establish more standardized research protocols and reporting criteria, such as unified cutoff values for hyperuricemia, standardized methods for uric acid measurement, consistent definitions and adjudication criteria for cardiovascular outcomes, and the use of standardized statistical models and covariates. Such efforts would reduce heterogeneity and facilitate a more reliable and persuasive elucidation of the relationship between hyperuricemia and CHD or other cardiovascular outcomes.* ……” Please review.

5. The term hyperuricemia is introduced early in the Introduction, but its definition (“elevated serum uric acid levels, a condition known as hyperuricemia”) only appears in the third paragraph. For clarity and better framing, the definition should be provided the first time the term appears, ideally in the opening paragraph, to ensure readers understand the concept from the outset.

Response: Thanks for your comment. This is an excellent suggestion to improve readability, and we acknowledge that we overlooked this point when drafting the manuscript. We have now revised the manuscript accordingly, that added the definition of hyperuricemia at its first appearance in the second paragraph in the Introduction section. Please review.

6. Several sentences are long and complex, which makes the manuscript difficult to read. Simplification and editing for concise scientific English are recommended. Topographical and grammatical errors should be corrected.

Response: Thanks for your suggestion. We have invited a researcher (Yan Li from the Yong Loo Lin School of Medicine, National University of Singapore) to help us revise the manuscript. We hope the quality of language has been improved with her help. Please review.

7. Abbreviations should be used consistently (e.g., CHD, CVD, MI).

Response: Thanks for this suggestion. We have carefully revised our manuscript to make sure the abbreviations were used consistently. Please review.

8. The authors are encouraged to ensure that all figures (including supplementary figures) are cited in the text in numerical order at the point of first mention. This will improve clarity and consistency for the reader.

Response: Thanks for your comment. We have carefully checked the manuscript, and found a typo at the first line of the “**Association between hyperuricemia and cardiovascular outcomes**” section. We have corrected the number of the figure, and checked the sequence of the other figures and tables (including the Supplementary figures and tables). Please review.

9. Supplementary Table S1 is dense and difficult to interpret. Organizing it by outcome type (CHD, CVD, MI) or by decade/region would improve readability.

Response: Thanks for your comment. Several articles involved more than 1 outcome; it is difficult for us to reorganize Table S1 by outcome type. To improve readability, in addition to adding new information (such as BMI and study initiation year) and adding the column heading to each page, we further streamlined the sections on uric acid measurement methods, outcome definitions, and adjusted factors, aiming to make Table S1 more concise and accessible. Because the table was revised in Excel and then pasted entirely into the Word document, specific track changes could not be displayed. We hope this will not cause any inconvenience during your review. Please kindly review it.

10. Figures S1–S30 present multiple subgroup and sensitivity analyses, but the number of studies (k) and participants (n) contributing to each analysis is not always clear. This information should be explicitly included in each figure legend. In addition, not all supplementary figures are cited in the manuscript text. For clarity and transparency, each figure should be referenced at least once, in sequential order, at the relevant point in the Results or Discussion.

Response: Thanks for the suggestion. To enhance the readability of the manuscript and the overall quality of the data presentation, we have provided as many figures as possible in the Supplementary Materials to support our findings. However, we believe that citing each supplementary figure individually within the main text would unnecessarily increase its length and reduce readability. Therefore, we reorganized the original 29 supplementary figures (previously Figure S2–S30) into two series according to the analysis groups: the hyperuricemia group and the per-unit increase in serum uric acid group, and reorganized them as the Figure S2 series (for hyperuricemia) and Figure S3 series (for per unit increase of uric acid), respectively. This approach allows us to present the results more clearly without compromising the readability of the manuscript. We also reviewed all relevant data and added the number of included studies and sample sizes in the legends of each supplementary figure. Please review.

Reviewer #3: This study is understood to be a systematic review and meta-analysis that examines the relationship between hyperuricemia and cardiovascular disease risk.

The research procedures, methodologies, interpretation of results, and discussion were conducted with great fidelity, including prior protocol registration, making this an exceptionally well-executed and high-quality study. A notable strength of the text is its comprehensive inclusion of recent research findings, which serves to enhance its credibility and relevance.

While the paper meets the standards for publication in an international journal at present, the following suggestions are offered to enhance its value further.

Response: Thank you for your valuable suggestions and for your recognition of our work. We have made substantial revisions to our manuscript based on your and the other reviewers’ comments, adding essential data and new analytical results. We hope these improvements have made our findings more comprehensive and robust. Please review.

Minor Point

A distinctive discovery from this study was the U-shaped association observed between serum uric acid levels and cardiovascular disease (CVD) mortality risk in men. In the conclusions, a discussion would be beneficial regarding physiologically plausible mechanisms that could explain why low uric acid levels are associated with an increased risk of cardiovascular disease (CVD) mortality.

Response: Thanks for your comment. We carefully reviewed the literature and attempted to interpret this finding. However, as presented in our newly added Supplementary Figure S4, a similar U-shaped association was also observed for CHD mortality in men (p>0.05), whereas the risk of CHD itself exhibited an inverted U-shaped association (p>0.05). These patterns were not observed among women. Although we explored potential explanations for the U-shaped association, we were unable to further clarify the reasons for the inverted U-shaped relationship for CHD risk in men, or why a similar pattern was not observed in women. We acknowledge this limitation in the Discussion section and emphasize that our findings are based on limited data, highlighting the need for further studies to validate these results. For this reason, this finding was not emphasized in the Conclusion section or the Abstract section. We also anticipate that future mechanistic investigations will provide a better understanding of the underlying causes of the U-shaped association. We have added the following text in the Discussion section: “*Our study identified a U-shaped association between serum UA levels and the risk of CVD mortality among men. As shown in Supplementary Figure S4, a similar U-shaped pattern was also observed for CHD mortality in men (p>0.05), whereas the risk of CHD itself demonstrated an inverted U-shaped association (p>0.05). Interestingly, these associations were not observed among women. Similar pattern has been observed from other studies as well, including kidney failure and mortality in chronic kidney disease[73], composite outcome of COVID-19 patients[74], etc. The relationship between hyperuricemia and endothelial dysfunction or vascular injury has been well recognized[75]. Some studies have suggested that uric acid possesses physiological antioxidant[76] and immunomodulatory properties[77], and that excessively low UA levels may impair free radical scavenging capacity and reduce immune regulation, ultimately leading to endothelial damage. This mechanism might explain the increased CVD mortality observed in men with low uric acid levels. However, it does not account for the inverted U-shaped association with CHD risk in men, nor the absence of similar patterns in women. It should be acknowledged that the number of original studies included in the dose-response meta-analysis was limited, which may affect the stability of the results. Further studies with high-quality are warranted to verify or correct our findings. We also anticipate that future research on uric acid will provide a more comprehensive understanding of its dual effects at different concentration levels.*” Please review.
